# Rb_4_CuSb_2_Cl_11_ and Rb_2_In_0.91(0.2)_Sb_0.09_Cl_5_·H_2_O: Wide Band Gap 0D Metal Halide Semiconductors

**DOI:** 10.1021/acs.inorgchem.5c04529

**Published:** 2025-12-16

**Authors:** Hamza Shoukat, Tamanna Pinky, Muhammad Sani Muhammad, Zaheer Masood, Arslan Akbar, Nobuyuki Yamamoto, Sharad Puri, David N. McIlroy, Bin Wang, Jakoah Brgoch, Bayram Saparov

**Affiliations:** † Department of Chemistry & Biochemistry, 6187 University of Oklahoma, Norman, Oklahoma 73019, USA; ‡ School of Sustainable Chemical, Biological and Materials Engineering, University of Oklahoma, Norman, Oklahoma 73019, United States; § Department of Chemistry and Texas Center for Superconductivity, 14743 University of Houston, Houston, Texas 77204, United States; ∥ Department of Physics, 7618 Oklahoma State University, Stillwater, Oklahoma 74078, United States

## Abstract

Herein, we report the discovery, structural and photophysical characterization of a new zero-dimensional (0D) lead-free all-inorganic halide, Rb_4_CuSb_2_Cl_11_, which adopts a new structure type. Single-crystal X-ray diffraction (SCXRD) shows that the structure consists of isolated, distorted seesaw [SbCl_4_]^−^ and trigonal planar [CuCl_3_]^2–^ units, separated by Rb^+^ cations that provide charge balance. Optoelectronic measurements and density functional theory (DFT) calculations indicate an indirect band gap of 2.89 eV, making it a candidate for wide-bandgap optoelectronic applications. Electrical resistivity was measured at 1.29 × 10^10^ Ω·cm, and the trap-state density (*n*_trap_) was found to be 7.44 × 10^10^ cm^–3^. Attempts to synthesize substitution analogs of Rb_4_CuSb_2_Cl_11_ led to the synthesis of Rb_2_In_0.91(0.2)_Sb_0.09_Cl_5_·H_2_O, which was erroneously reported as Rb_2_SbCl_5_O in a previous study. Rb_2_In_0.91(0.2)_Sb_0.09_Cl_5_·H_2_O adopts a vacancy-ordered perovskite structure and exhibits broad-band yellow emission under UV excitation. The measured photoluminescence quantum yield (PLQY) for Rb_2_In_0.91(0.2)_Sb_0.09_Cl_5_·H_2_O is 18.2%. These findings add to the growing class of quaternary metal halides with multiple cation and anion compositions, expanding the chemical phase space for the discovery of new materials with functional properties.

## Graphical abstract

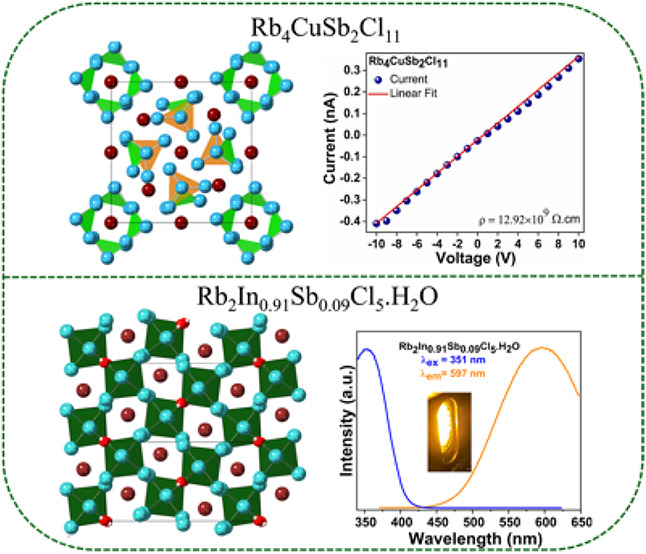

## 1 Introduction

Metal halide perovskites have attracted considerable attention over the past two decades following the discovery of lead halide perovskites as high efficiency energy materials for solar energy conversion.^1,2^ The exceptional optoelectronic properties of these compounds have driven extensive research worldwide, leading to the exploration of halide perovskites for applications beyond solar including in light emission, radiation detection, and ferroelectric devices.^3–8^ Central to these advances is the unique structural versatility of perovskites, which enables fine-tuning of their electronic and optical properties. Researchers define perovskites based on their resemblance to the mineral perovskite crystal structure, described by the general formula ABX_3_ for the three-dimensional (3D) perovskites.^9^ In the ABX_3_ perovskite structure, the BX_6_ octahedral building blocks are connected through corner-sharing in all three crystallographic directions. Despite their outstanding photophysical properties and optoelectronic device performances, conventional lead-based perovskites face significant challenges related to toxicity and long-term stability. The toxicity and poor environmental stability of Pb­(II)-based perovskites have motivated intense research on lead-free metal halide alternatives.^10,11^ Strategies include replacing Pb­(II) with other divalent cations such as Sn­(II) and Ge­(II) or introducing pairs of monovalent (M­(I)) and trivalent (M­(III)) cations to form so-called double perovskites.^12–17^ A range of these double perovskite compositions have since been reported, broadening the design space for lead-free halide materials with potential for optoelectronic applications; examples include, but are not limited to Cs_2_AgBX_6_ (*B* = Bi, Tl, In, *X* = Cl, Br),^18^ Cs_4_Au^II^Au^III^_2_Cl_12_,^19^ Cs_4_CuSb_2_Cl_12_,^20^ (CHA)_4_CuBiI_8_,^21^ (CHA = cycloheptylammonium), (PEA)_4_CuInCl_8_ (PEA = phenethylammonium),^22^ (C_6_N_2_H_14_)_2_SbCuCl_9_·2H_2_O^23^ and *A*(*L*)_*n*_[*B*Cl_*m*_] (*A* = rare earth (RE), alkaline earth metals and Mn; *L* = solvent ligand; and *B* = Sb, Bi and Te).^24^

Beyond the conventional corner-sharing or edge-sharing networks found in metal halide perovskites and their structural derivatives, a class of 0D organic–inorganic dual-metal halides has also emerged. These materials typically consist of an A-site cation, two distinct metal centers (M_*x*_ and M_*y*_), and halide anions, but lack extended octahedral connectivity.^25^ As a result, they fall outside the traditional definition of halide perovskites, and instead, form isolated polyanionic clusters that are stabilized in 0D architectures. Several examples of such systems have been reported in the literature, including (bmpy)_9_[Pb_3_Br_11_]­(MnBr_4_)_2_, (bmpy)_9_[SbCl_5_]_2_[Pb_3_Cl_11_], (Bmpip)_2_Pb_*x*_Sn_1–*x*_Br_4_, and (bmpy)_9_[ZnCl_4_]_2_[Pb_3_Cl_11_].^26–29^ In these cases, the 0D polyanionic subunits are stabilized by bulky organic cations, which introduce lattice softness and spatial flexibility to accommodate diverse polyhedral geometries.^30^

Herein, we introduce a new member of this growing family—Rb_4_CuSb_2_Cl_11_, which is an all-inorganic 0D metal halide semiconductor. This compound is structurally distinct from traditional double perovskites as it lacks octahedral building blocks and does not form an extended 3D framework. Instead, the structure of Rb_4_CuSb_2_Cl_11_ comprises two distinct polyanions, seesaw [SbCl_4_]^−^ and trigonal planar [CuCl_3_]^−^, which are isolated by Rb^+^ cations. Notably, this is the first report of a 0D structure containing two metal halide polyanions stabilized by relatively small Rb^+^ ions as opposed to prior reports of structures stabilized by larger organic cations. This finding expands the design space for targeted preparation of low-dimensional metal halides. Additional synthesis experiments targeting a substitutional analog Rb_4_CuSb_*x*_In_1–*x*_Cl_11_ resulted in the isolation of crystals of Rb_2_In_0.91(0.2)_Sb_0.09_Cl_5_·H_2_O. Interestingly, this compound was obtained under comparable synthetic conditions to that in a previous study, however, its composition was reported as Rb_2_SbCl_5_O.^31^ Upon careful further investigation, our results reveal a different composition than the earlier report. Comprehensive characterization through X-ray photoelectron spectroscopy (XPS), scanning electron microscopy and energy-dispersive X-ray spectroscopy (SEM–EDS), and Fourier-transform infrared (FTIR) spectroscopy confirmed the formation of an Sb-doped indium chloride phase, Rb_2_In_0.91(0.2)_Sb_0.09_Cl_5_·H_2_O, featuring a coordinated water molecule within the lattice. In this study, we further characterize photophysical properties of Rb_4_CuSb_2_Cl_11_ and Rb_2_In_0.91(0.2)_Sb_0.09_Cl_5_·H_2_O, which are discussed together with the results of DFT calculation results reported for the first time.

## 2 Experimental Section

### 2.1 Materials

Antimony­(III) chloride (99.90%, Aldrich), hydrochloric acid (37%, Sigma-Aldrich), copper­(I) chloride (99%, Thermo Scientific), rubidium chloride (99.8%, Acros Organics), indium­(III) chloride (Aldrich), isopropanol (Fisher), and hypophosphorous acid (H_2_PO_3_) (50%, Sigma-Aldrich), were purchased and used with no further purification, unless otherwise stated. All synthesis experiments were performed in a fume hood under standard conditions.

### 2.2 Synthesis of Rb_4_CuSb_2_Cl_11_ Single Crystals

Stoichiometric amounts of RbCl, CuCl and SbCl_3_ were combined in a 4:1:2 ratio (in mmol scale) inside a nitrogen-filled dry glovebox. The reactants were placed into a 20 mL glass vial, sealed with a rubber septum prior to removal from the glovebox to prevent moisture exposure. Outside the glovebox, 6 mL of concentrated HCl (37%) was carefully added to the vial, followed by the addition of 0.2 mL of hypophosphorous acid H_2_PO_3_ as a reducing agent to prevent the oxidation of Cu­(I). The reaction mixture was stirred on a hot plate for 1 h to ensure complete dissolution and homogenization. Subsequently, the vial was placed in an oil bath and gradually heated to 95 °C with a closed cap, with continued stirring until a saturated solution was formed. Crystal growth was achieved by applying a cooling-induced crystallization method; the saturated solution was slowly cooled to room temperature, leading to the formation of light-yellow block-shaped crystals of the target compound. The obtained crystals were washed with isopropanol, dried under vacuum, and stored in the glovebox for further characterization. By carefully controlling the cooling rate, it was possible to grow larger and higher-quality crystals; a cooling rate of approximately 1 °C per hour proved optimal for producing centimeter-sized single crystals.

### 2.3 Synthesis of Rb_2_In_0.91(0.2)_Sb_0.09_Cl_5_·H_2_O Single Crystals

The synthesis of Rb_2_In_0.91(0.2)_Sb_0.09_Cl_5_·H_2_O is particularly interesting, as it can be obtained under certain conditions only. The compound was first isolated during an attempt to substitute Sb with In in Rb_4_CuSb_2_Cl_11_. In this process, RbCl (4 mmol), CuCl (1 mmol), SbCl_3_ (1 mmol) and InCl_3_ (1 mmol) were dissolved in 10 mL of concentrated HCl, 0.02 mL of H_2_PO_3_ was added as a reducing agent, and the solution was heated to 110 °C, followed by slow cooling to room temperature at a rate of 1 °C per hour. This procedure led to the isolation of small, transparent crystals of Rb_2_In_0.91(0.2)_Sb_0.09_Cl_5_·H_2_O.

### 2.4 Powder X-ray Diffraction (PXRD)

PXRD measurements were performed at room temperature using a Rigaku MiniFlex600 diffractometer equipped with Ni-filtered Cu Kα radiation. Data were collected over the 2θ range of 2° to 90°, with a step size of 0.02°. The resulting diffraction patterns were processed and analyzed using the PDXL2 software suite.

### 2.5 Single Crystal X-ray Diffraction (SCXRD)

SCXRD data were collected using a Bruker D8 Quest diffractometer equipped with a Kappa goniometer, an Incoatec IμS microfocus Mo Kα X-ray source, and a Photon II area detector. Absorption corrections were applied using a semiempirical method based on equivalent reflections, as implemented in the APEX3 v2015.5–2 software suite. The crystal structures were solved by intrinsic phasing, and site occupancy factors were carefully evaluated by allowing the occupancies of each unique crystallographic site to refine freely. Detailed data collection parameters and crystallographic information are summarized in Table 1, while atomic coordinates, equivalent isotropic displacement parameters, and selected bond lengths and angles are presented in Tables S1–S3.^32–35^ The corresponding Crystallographic Information Files (CIFs) have been deposited in the Cambridge Crystallographic Data Centre (CCDC) under deposition numbers (2481828 and 2501691).

**Table 1:** Selected Single Crystal Data and Structure Refinement Parameters for Rb_4_CuSb_2_Cl_11_ and Rb_2_In_0.91(0.2)_Sb_0.09_Cl_5_·H_2_O^a^

Compound	Rb_4_CuSb_2_Cl_11_	Rb_2_In_0.91(0.2)_Sb_0.09_Cl_5_·H_2_O
Formula weight(g/mol)	1038.87	481.65
Crystal system	Tetragonal	Orthorhombic
Space group	*P*4_**2**_/*m*	*Pnma* (62)
*Z*	4	4
Unit cell dimensions (Å)	*a* = 13.7455(6) *c* = 11.2139(8)	*a* = 14.053(6) *b* = 10.192(5) *c* = 7.263(3)
Volume (Å^3^)	2118.7(2)	1040.3(8)
Density (ρ_calc_) (mg/m^3^)	3.257	3.075
θ_min_ – θ_max_ (°)	2.095 to 26.731°	2.899 to 25.020°
Absorption coefficient (μ) (mm^–1^)	14.023	12.821
Reflections collected	21210	4915
Independent reflections	2365 [*R*_int_ = 0.0815]	943 [*R*_int_ = 0.0713]
Completeness to θ= 25.242°	99.4%	96.4%
*R* ^*a*^ indices (*I* > 2σ(*I*))	*R*_1_ = 0.0499, w*R*_2_ = 0.1129	*R*_1_ = 0.0641, w*R*_2_ = 0.1536
Goodness-of-fit on *F* ^2^	1.259	1.086
Largest diff. peak and hole (e/Å^3^)	1.595 and −3.207	2.599 and −2.179

a ${R_{1}=\sum \left|\left|F_{0}\right|-\left|F_{c}\right|\right|/\sum \left|F_{0}\right|;wR_{2}=\left|\Sigma \right|w{\left(F_{0}^{2}-F_{c}^{2}\right)}^{2}/\sum |wF_{0}^{2^{2}}|}^{1/2}$

where $w=1/\left|\sigma^{2}F_{0}^{2}+{\left(AP\right)}^{2}+BP\right|$ , with $P=\left(F_{0}^{2}+2F_{c}^{2}\right)/3$ and weight coefficients *A* and *B.*

### 2.6 Thermogravimetric Analysis (TGA) and Differential Scanning Calorimetry (DSC)

TGA and DSC measurements were performed on 5–8 mg samples using a TA Instruments SDT 650 thermal analyzer system. Crystals were heated from 25 to 475 °C under a nitrogen gas flow at a rate of 100 mL/min and a heating rate of 5 °C/min. Melting point measurements for Rb_4_CuSb_2_Cl_11_ were taken on a Mel-Temp apparatus (110/120VAC; 50/60 Hz and 200 W). The heating element was initially set at 50 V and later increased to 60 V. Measurements took approximately (5 h), using capillary tubes (0.8–1.2 × 90 mm).

### 2.7 Photoluminescence Measurements

Room-temperature photoluminescence (PL) and photoluminescence excitation (PLE) spectra were recorded on single crystals of both semiconductors using a HORIBA Jobin Yvon Fluorolog-3 spectrofluorometer, equipped with a xenon lamp as the excitation source and a Quanta-Φ integrating sphere for absolute quantum yield measurements. Data were collected over the 250–750 nm spectral range using the two-curve method. For determination of the PLQY, the samples were placed inside the Quanta-Φ integrating sphere attached to the Fluorolog-3 system. Measurements were performed by exciting the crystals at their respective photoluminescence excitation maxima, under full lamp power, to ensure optimal emission detection.

### 2.8 Diffuse Reflectance Measurements

UV–Vis diffuse reflectance spectra were collected on gently ground powder samples using a PerkinElmer LAMBDA 750 UV–Vis–NIR spectrometer, equipped with a 100 mm InGaAs integrating sphere, covering the spectral range from 250 to 1100 nm. The reflectance data were converted into pseudoabsorption spectra by applying the Kubelka–Munk function, from which the optical band gaps were subsequently estimated.

### 2.9 Computational Studies

All DFT calculations were performed using the Vienna Ab-initio Simulation Package (VASP).^36^ Initial structures were obtained from experimental CIF files and subsequently optimized using the Perdew–Burke–Ernzerhof (PBE) exchange-correlation functional^37^ within the generalized gradient approximation (GGA), along with projector augmented wave (PAW) potentials.^38,39^ Geometry optimizations were carried out using either the quasi-Newton scheme or the conjugate gradient algorithm with a plane-wave energy cutoff of 400 eV, until the forces on each atom were reduced below 0.02 eV Å^–1^ and the total energy converged to less than 10^–6^ eV. Gaussian smearing with a width of 0.02 eV was applied. To account for van der Waals interactions, Grimme’s DFT-D3 dispersion correction with zero damping was employed.^40,41^ The Brillouin zone was sampled using a Γ-centered 6 × 6 × 6 k-point mesh for Rb_4_CuSb_2_Cl_11_ and a 3 × 3 × 5 mesh for Rb_2_InCl_5_·H_2_O and Rb_2_SbCl_5_·H_2_O. On the PBE-optimized geometries, band gap calculations were performed using the HSE06 hybrid functional.^42^ The electronic convergence criterion was set to 10^–6^ eV. Pre- and postprocessing, including k-path generation and density of states (DOS) analysis, were conducted using VASPKIT.^43^

### 2.10 Electrical Measurements and X-ray Response Tests

To perform electrical measurements and evaluate the X-ray response characteristics, a prototype X-ray detector was fabricated by applying high-quality silver paste (Ted Pella, Inc.) to the opposite faces of a single crystal of Rb_4_CuSb_2_Cl_11_. A Keithley 6487 pico-ammeter was employed for current–voltage (*I*–*V*) and space-charge-limited current (SCLC) measurements. X-ray response measurement was conducted by using a Rigaku MicroMax 007HF microfocus X-ray generator, which is equipped with a Cu target, and the detector was exposed to 8 keV soft X-rays. The X-ray dose rate was carefully calibrated using a commercial dosimeter.

### 2.11 Temperature-Dependent Photoluminescence Measurements

Temperature-dependent photoluminescence studies were conducted using a Janis Cryostat (VPF-100) to control the environment from 80 to 300 K, on PTI Quanta Master 400 equipped with a 75 W Xe steady-state lamp (PTI Instruments). The sample was placed in the cryostat under vacuum and cooled to 80 K, afterward sample was excited at 351 nm, and emission spectra were collected on heating.

### 2.12 Scanning Electron Microscopy and Energy-Dispersive X-ray Spectroscopy (SEM–EDS)

Morphological and compositional analyses were performed using a Thermo Fisher Scientific (formerly FEI) Apreo/AXIS field-emission scanning electron microscope (SEM) equipped with an energy-dispersive X-ray spectroscopy (EDS) detector. The samples were mounted on aluminum stubs using conductive carbon tape and sputter-coated with a thin layer of gold (∼5 nm) to minimize surface charging. SEM images were acquired at an accelerating voltage of 15 kV and a working distance of 10 mm under high-vacuum conditions. Elemental mapping and point analysis were conducted using an Oxford Instruments EDS system integrated with Thermo Fisher’s Avizo/EDAX software. Quantitative results were obtained from the atomic and weight percentages calculated using ZAF (atomic number, absorption, and fluorescence) correction.

### 2.13 X-ray Photoelectron Spectroscopy (XPS)

The XPS spectra of samples were obtained using the Mg Kα X-ray emission line from a dual-anode X-ray source (PREVAC XR40B-EC) in a custom-built ultrahigh vacuum chamber with a base pressure of 5.0 × 10–10 Torr operated at 345 W. The kinetic energy of the photoelectrons was measured with an Omicron EA 125 hemispherical electron energy analyzer (Melbourne, VIC, Australia) with a resolution of 0.01 eV.

### 2.14 Fourier Transform Infrared Spectroscopy (FTIR)

Fourier transform infrared (FTIR) spectra were recorded using a Thermo Nicolet Avatar spectrometer equipped with a diamond attenuated total reflectance (ATR) accessory. The measurements were performed in the range of 4000–400 cm^–1^ with a spectral resolution of 4 cm^–1^, and each spectrum was averaged over 32 scans to improve the signal-to-noise ratio. Sample was analyzed in solid state without further preparation by placing them directly on the diamond crystal.

## 3 Results and Discussion

Large, block-shaped crystals of Rb_4_CuSb_2_Cl_11_ can be synthesized by cooling-induced crystallization of a saturated solution. The selected cooling rate impacts the size of obtained crystals (see Section 2.2). The phase purity and crystallinity of the obtained sample have been verified through PXRD experiments (Figure S1).

**Figure 1:**
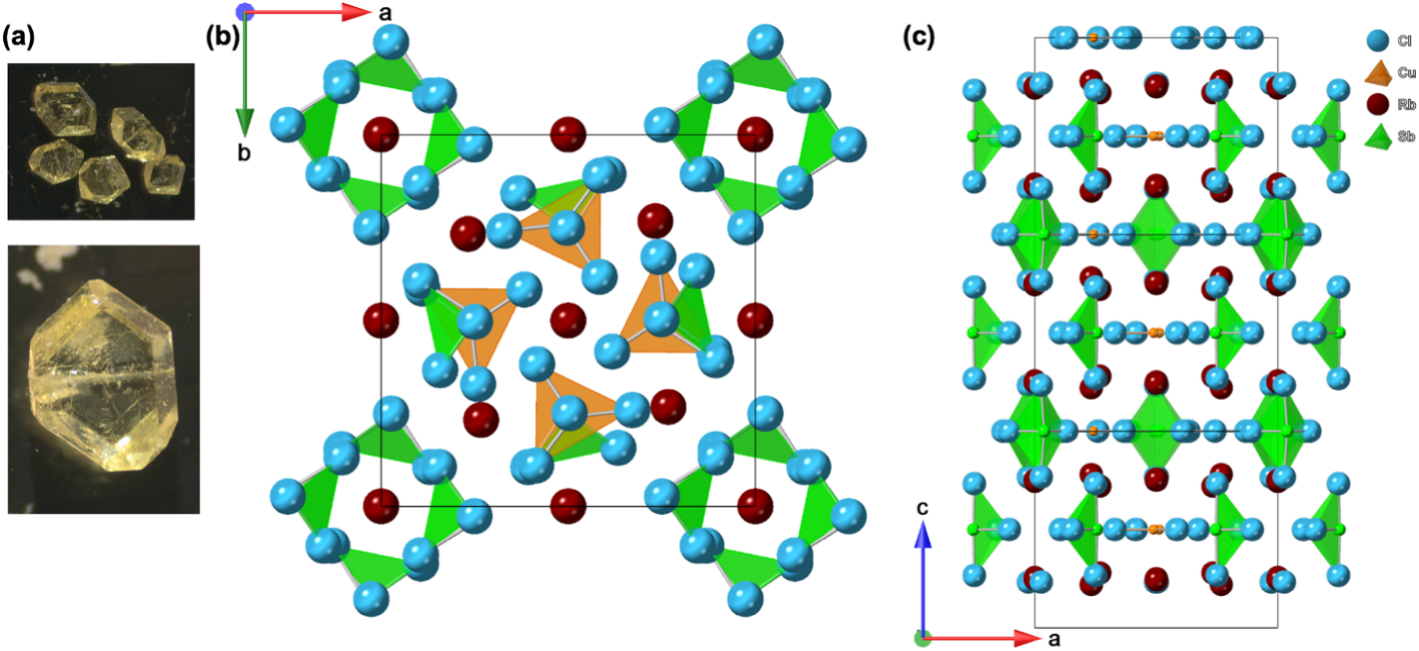
(a) Pictures of block-shaped Rb_4_CuSb_2_Cl_11_ crystals. (b–c) Polyhedral representations of the crystal structure of Rb_4_CuSb_2_Cl_11_ along different axes.

SCXRD experiments suggest that Rb_4_CuSb_2_Cl_11_ crystallizes in the tetragonal crystal system with the centrosymmetric space group *P*4_2_/*m* (Table 1) with 16 crystallographically unique atoms within the asymmetric unit. The presence of multiple *B*-metal centers in Rb_4_CuSb_2_Cl_11_ leads to the distinct coordination polyhedra around Cu and Sb. Thus, the structure of Rb_4_CuSb_2_Cl_11_ is comprised of trigonal planar [CuCl_3_]^2–^ and seesaw [SbCl_4_]^−^ anions that are separated by Rb^+^ cations (Figure 1). The crystal structure consists of discrete, well-separated polyhedral building blocks, which are periodically repeated to form the 0D lattice. Analysis of the crystallographic data reveals that the separation of [SbCl_4_]^−^ from each other is at ∼3.6 Å as judged by the nearest distances between chloride ions on the adjacent seesaw units (Figure S2). In contrast, trigonal planar [CuCl_3_]^2–^ are spaced at a longer distance of 3.836 Å from one another. Finally, the nearest chloride distances between the dissimilar [CuCl_3_]^2–^ and [SbCl_4_]^−^ range from 3.576 to 3.931 Å (Figure S2). The observed distances between chlorides in adjacent [CuCl_3_]^2–^ and [SbCl_4_]^−^ polyanions as well as distances between the neighboring [SbCl_4_]^−^ anions are comparable to the expected distance between closely packed chloride anions (1.81 Å × 2 = 3.62 Å).^44^ This is an important observation suggesting that although Rb_4_CuSb_2_Cl_11_ has a 0D crystal structure as judged by the presence of isolated molecular anions, close packing of anions in Rb_4_CuSb_2_Cl_11_ may lead to increased charge delocalization for a structurally 0D compound. In turn, this could have important implications for its optoelectronic properties.^45^

The presence of distorted seesaw [SbCl_4_]^−^ units in the structure of Rb_4_CuSb_2_Cl_11_ can be attributed to the stereochemically active lone pairs on Sb­(III); the Cl–Sb–Cl bond angles deviate from 90 and 180°, with respective angles ranging from 85.65 to 91.14(5)^o^ and 170.95° (Figure S3).^46^ Correspondingly, the Sb–Cl bond lengths in this 4-coordinate environment vary between 2.396–2.625 Å, reflecting the distortion induced by the lone pairs on Sb­(III). Additionally, there is another chloride at a longer Sb···Cl distance of 2.832 Å, inclusion of which into the coordination environment of Sb­(III) leads to a five-coordinated square pyramidal geometry (Figure S4). However, while the Sb–Cl bonding distance of 2.396–2.625 Å is within the normal range,^47^ the distance of 2.832 Å is too long to be considered a typical polar covalent Sb–Cl bond. Nevertheless, the presence of this elongated contact to a neighboring [CuCl_3_]^2–^ unit also leads to the distortion of the latter. In the trigonal planar [CuCl_3_]^2–^, distorted Cl–Cu–Cl bond angles range from 109–133°, deviating from the ideal 120° (Figure S3), due to the influence of the adjacent [SbCl_4_]^−^ units. Note that the issue of Sb­(III) coordination could also be important for determining the light emission behavior of this material: compounds containing [SbCl_5_]^2–^ units are known to be strongly photoluminescent, exhibiting bright yellow-orange emission, whereas Rb_4_CuSb_2_Cl_11_ shows quenched PL.^48^

To further describe this new structure type, which deviates from the conventional double perovskite structure, we carefully examined the coordination environments of the Rb^+^ cations. Rb_4_CuSb_2_Cl_11_ features four crystallographically distinct Rb sites (Figure S5). In 3D halide perovskites, the larger A-site cation has a 12-fold cuboctahedral coordination,^2^ whereas in Rb_4_CuSb_2_Cl_11_, coordination numbers of Rb sites range between 8–12. Rb1 is coordinated to 12 chlorine atoms in a nearly ideal cuboctahedral geometry. Both Rb2 and Rb4 are 8-coordinated, but with distinct distortions: Rb2 has a distorted cuboidal environment, whereas Rb4 coordination polyhedron more closely resembles a distorted dodecahedron. The Rb3 site, with its 9-fold coordination, adopts a distorted capped square antiprismatic geometry (Figure S5). These variations in the Rb coordination highlight the structural flexibility of the lattice in accommodating *A* cations of within different local environments. Such lower coordination number for the *A* cation is uncommon in ternary metal halide systems, therefore, this observation also highlights the structural flexibility and adaptive nature of Rb^+^ in metal halides.^49–52^

Transparent, block-shaped crystals of Rb_2_In_0.91(0.2)_Sb_0.09_Cl_5_·H_2_O were obtained when attempting to replace Sb with In in Rb_4_CuSb_2_Cl_11_. The formation of Rb_2_In_0.91(0.2)_Sb_0.09_Cl_5_·H_2_O is particularly interesting, as the initial SC-XRD refinement suggested a composition of Rb_2_SbCl_5_O, closely matching a previously reported structure.^31^ In the prior report, this material was obtained as an impurity phase and In­(III) was present in the reaction mixture. However, despite several synthetic attempts, we were unable to reproduce the reported Rb_2_SbCl_5_O, which suggested a potential incorporation of In­(III) into the structure and the presence of water in the structure instead of an oxide anion, i.e., a composition of Rb_2_(In,Sb)­Cl_5_·H_2_O. This observation aligns with the fact that Rb_2_InCl_5_·H_2_O is a known compound.^53^ The difficulty in stabilizing the fully substituted Sb Rb_2_SbCl_5_·H_2_O analog may stem from the stereochemically active 5s^2^ lone pair on Sb^3+^, which resists incorporation into the compact vacancy-ordered perovskite framework, favoring instead dimeric coordination environments.^54^

To verify the elemental composition and oxidation states of the constituent metals, XPS measurements were performed on both Rb_4_CuSb_2_Cl_11_ and Rb_2_In_0.91(0.2)_Sb_0.09_Cl_5_.H_2_O. In Rb_4_CuSb_2_Cl_11_, distinct peaks corresponding to Rb 3d, Cu 2p, and Sb 3d_5/2_–3d_3/2_ were observed, with binding energies around 529 and 539 eV. These features confirm the presence of Cu^+^ and Sb^3+^ oxidation states (Figure S10).^55^ The XPS spectrum of Rb_2_In_0.91(0.2)_Sb_0.09_Cl_5_·H_2_O is particularly noteworthy, displaying a pronounced In 3d_5/2_ peak at approximately 445 eV, consistent with In^3+^.^56^ The Sb 3d region in this compound similarly indicates Sb^3+^ oxidation, corroborating the mixed In/Sb composition determined from the SCXRD (Figure S11). This is further supported by the SEM–EDS measurements (Figure S12), which suggest an average composition of Rb_2.06_In_1.00_Sb_0.07_Cl_4.37_·(H_2_O)_2.5_. The deviation of the SEM–EDS estimated composition from the SCXRD results is due to the low Sb doping content and the expected elevated estimation of oxygen content due to the presence of crystal surface oxygens. Finally, FTIR spectroscopy measurements were conducted to confirm the presence of coordinated water molecules in Rb_2_In_0.91(0.2)_Sb_0.09_Cl_5_·H_2_O. Two characteristic vibrational bands corresponding to O–H stretching and bending modes were observed at approximately 3450 cm^–1^ and 1650 cm^–1^, respectively (Figure S13). Collectively, these results support the revised chemical formula of the compound as Rb_2_In_0.91(0.2)_Sb_0.09_Cl_5_·H_2_O.

The obtained crystals of Rb_2_In_0.91(0.2)_Sb_0.09_Cl_5_·H_2_O are of high quality as evidenced by our PXRD and SCXRD measurement results (Figure S1 and Table 1). Rb_2_In_0.91(0.2)_Sb_0.09_Cl_5_·H_2_O crystallizes in the centrosymmetric space group *Pnma* (62) (Table 1), isostructural with the parent Rb_2_InCl_5_·H_2_O, with indium and antimony sharing the same crystallographic site (Figure 2). Refinement of the mixed occupancy indicated a dominant contribution from In^3+^ with a minor substitution by Sb^3+^ (≈9%), suggesting a limited solubility of Sb^3+^ in this structure. The structure is derived from the well-known vacancy-ordered perovskite structure, i.e., the cubic K_2_PtCl_6_-type (*Fm*-3*m*) structure, members of which include Cs_2_SnI_6_ and Cs_2_TeI_6_.^16,57^ However, the symmetry of Rb_2_In_0.91(0.2)_Sb_0.09_Cl_5_·H_2_O is reduced due to the presence of multiple anions. In Rb_2_In_0.91(0.2)_Sb_0.09_Cl_5_·H_2_O, there is a single trivalent metal M site in the center of an octahedron surrounded by 5 chloride anions and 1 water as a coordinated ligand; these octahedra are separated by Rb^+^ cations to form the 0D structure of Rb_2_In_0.91(0.2)_Sb_0.09_Cl_5_·H_2_O. The substitution appears to introduce subtle local distortions due to the stereochemically active lone pair of Sb^3+^. Within the [(In,Sb)­Cl_5_H_2_O]^2–^ octahedra, the shorter M–O bond (2.244 Å) introduces significant octahedral distortion, with the remaining M–Cl bonds are in the 2.479–2.514 Å range consistent with the previous report (Table S3 and Figure S6).^53^ Despite their 0D crystal structures, K_2_PtCl_6_-type metal halides are known to feature closely packed anionic lattices.^58^ The closest Cl···Cl distance in the neighboring [(In,Sb)­Cl_5_H_2_O]^2–^ octahedra is 3.435 Å, indicating the close packing of the chloride anionic lattice in Rb_2_In_0.91(0.2)_Sb_0.09_Cl_5_·H_2_O (Figure 2).

**Figure 2:**
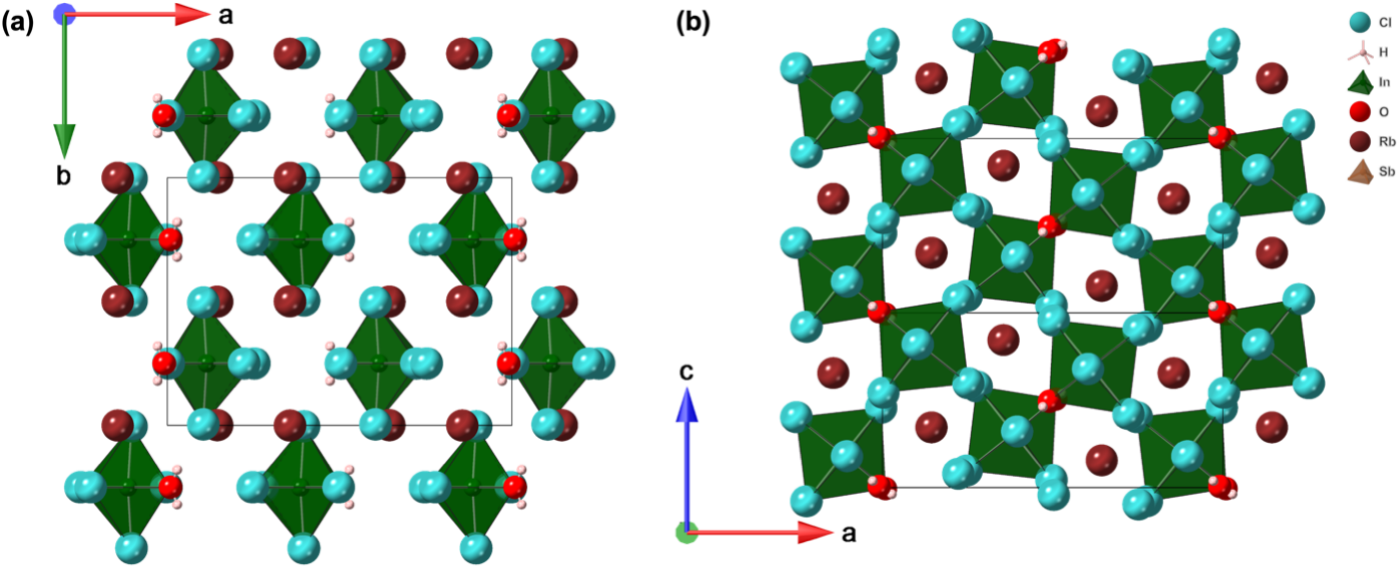
Polyhedral representations of the 0D vacancy-ordered perovskite crystal structure of Rb_2_In_0.91(0.2)_Sb_0.09_Cl_5_·H_2_O viewed down the (a) *a*- and (b) *b*-axes.

To determine the optical properties, we performed photophysical characterization of the material using diffuse reflectance and PL spectroscopies. The diffuse reflectance data for Rb_4_CuSb_2_Cl_11_ were converted into pseudoabsorbance using the Kubelka–Munk function, F(R)=αS=(1−R)2/2R , where *R* is the reflectance, α is the absorption coefficient, and *S* is the scattering coefficient (Figure 3a).^52^ The *F*(*R*) plot shows a well-defined absorption onset at 2.89 eV. The Tauc plots derived from the diffuse reflectance data (Figure S7) assuming indirect and direct optical band gaps yield values of 2.78 and 3.05 eV, respectively. Such high band gap values for Rb_4_CuSb_2_Cl_11_ suggests a wide band gap semiconductor behavior, as is expected for a low dimensional metal chloride compound.^59^ It is worth noting that the band gap of a related compound, Cs_4_CuSb_2_Cl_12_, is reported to be around 1.0 eV. The earth-abundant elemental composition of this material and its much lower band gap suitable for photovoltaic applications attracted interests of researchers.^20^ The key difference between Cs_4_CuSb_2_Cl_12_ and Rb_4_CuSb_2_Cl_11_ is the Cu oxidation state, which is +2 in the former and +1 in the latter. The [Ar] 3d^10^ electronic configuration of Cu­(I) in Rb_4_CuSb_2_Cl_11_ not only results in the absence of *d*–*d* transitions in the optical absorption spectra but also leads to the smaller coordination number of 3 for Cu­(I) (vs octahedral (CN = 6) coordination around Cu­(II) in Cs_4_CuSb_2_Cl_12_). Theoretical studies on model structures suggest that while 3- and 4-fold coordinations are favored for Cu­(I), 6-fold coordination is unlikely due to the its small size and the high energy level of Cu 3*d* orbitals.^60^ In combination, these two factors result in the preference of low coordination numbers around Cu­(I) to allow for sufficient *d*–*s* and *d*–*p* hybridization.

**Figure 3:**
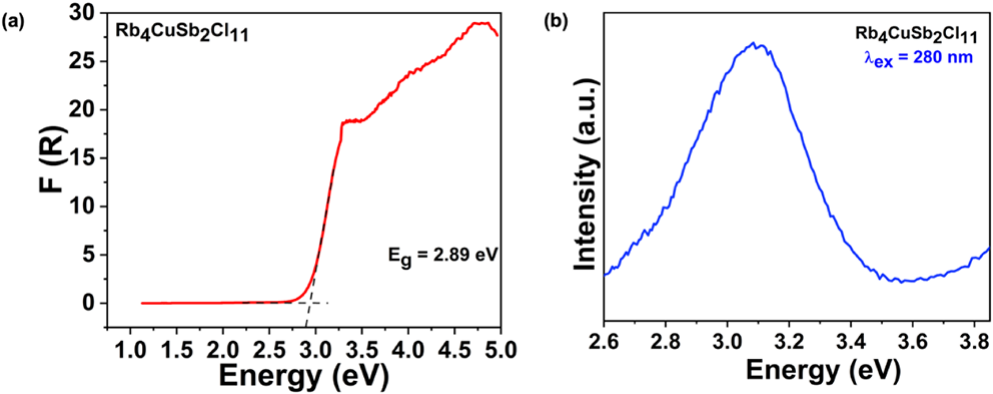
(a) Diffuse reflectance spectrum converted to *F*(*R*) plotted against photon energy. (b) Room temperature PL spectrum shows a broadband emission for Rb_4_CuSb_2_Cl_11_ under UV irradiation (280 nm).

Although Rb_4_CuSb_2_Cl_11_ does not show any visible emission under UV irradiation (e.g., under a UV lamp), we performed PL measurements to gain a deeper understanding of its photophysical properties. It appeared that under high-energy excitation at 280 nm, the material shows very weak emission with a full width at half-maximum (fwhm) around 400 nm; half of the peak lies in the UV region, spanning roughly 2.0–3.1 eV (Figure 3b). The observed emission seems to originate from band-edge emission. This emission is weak because of multiple contributing factors including the indirect band gap of Rb_4_CuSb_2_Cl_11_ (see the computational results below), similar to the weak emission observed for the related indirect band gap metal halide Rb_4_Ag_2_BiBr_9_.^52^ Second, although the anionic building blocks in Rb_4_CuSb_2_Cl_11_ are isolated, the halide anions are closely packed, leading to a higher degree of electronic dimensionality and charge delocalization. Therefore, Rb_4_CuSb_2_Cl_11_ cannot be directly compared to high-efficiency light emitting molecular Sb­(III) and Cu­(I) halides in which [*M*_*a*_ *X*_*b*_]^*c*^– (*M* = Sb­(III) or Cu­(I); *X* = Cl^–^, Br^–^, I^–^) are well-separated by larger organic cations.^2^ Finally, the unusual band structure of Rb_4_CuSb_2_Cl_11_ could also explain the quenched PL; the band structure of the compound indicates that the valence band is primarily derived from Cu­(I) orbitals and the conduction band is dominated by Sb­(III). This suggests that a charge separation could occur upon excitation (Cu­(I) → Sb­(III)), separating electrons and holes on distinct structural units and potentially leading to nonradiative decay. Notably, the [SbCl_4_]^−^ units adopt a seesaw geometry in Rb_4_CuSb_2_Cl_11_, which are often nonluminescent due to parity-forbidden transitions.^47,61^

The Tauc plots derived from the diffuse reflectance data (Figure S8) assuming indirect and direct optical band gaps for Rb_2_In_0.91(0.2)_Sb_0.09_Cl_5_·H_2_O give values of 2.95 and 3.29 eV, respectively. These band gap values are consistent with that reported for the other Sb-doped In halides.^53^ Since the crystals of Rb_2_In_0.91(0.2)_Sb_0.09_Cl_5_·H_2_O emit intense yellow light under a UV lamp, PL was performed to further characterize its optical properties. Upon 351 nm excitation, a broadband emission peak centered at 597 nm was observed; the difference between the two gives a remarkably large Stokes shift of 246 nm. While the parent Rb_2_InCl_5_·H_2_O is not luminescent due to parity forbidden transitions, Sb^3+^ doping unlocks the emission due to the allowed ^3^P_1_ to ^1^S_0_ transition,^62^ which has also been reported for other antimony halides.^63–65^

Previous studies on the Sb^3+^ doped In halides have shown that doping enhances emission only up to an optimal level, with PLQY rising to nearly 100% at ∼3–4% Sb. Beyond this point, however, further substitution leads to concentration quenching, as increased Sb–Sb interactions promote exciton migration to nonradiative sites, causing the PLQY to decline sharply. At higher dopant levels (≥7–8% Sb), the nonradiative decay rate increases significantly and the PLQY begins to drop. Since our crystals contain ∼9% Sb, it naturally falls within this quenching regime, explaining its moderate PLQY of ∼18%. This behavior is fully consistent with the established concentration-dependent emission trend in Sb-doped In-based halides.^62^

To further explore the emission mechanism, excitation-dependent PL and emission-dependent PLE and temperature-dependent PL measurements were performed (Figure 4 and Figure S9). The emission profile remained unchanged when excitation energy is varied from low to high, which supports the STE-based emission mechanism. Temperature-dependent PL measurements are particularly interesting, as upon cooling from 300 to 220 K, the emission intensity gradually decreases, accompanied by the emergence of a shoulder peak centered around 510 nm (Figure S9). Further lowering the temperature leads to a steady increase in overall emission intensity, and at 80 K, the combined intensity of the two peaks becomes comparable to that observed at 220 K for the single-emission feature. This antithermal quenching can be related to the participation of shallow trapped states arising due to structural defects.^66^ Similar temperature-dependent dual-emission behavior has been observed in other Sb-based halides, where the interplay between lattice relaxation and exciton self-trapping governs the emission process.^67^ Finally, to rule out surface defect-related emission, PL spectra were measured for both single crystals and powder samples; the resultant PL spectra are similar, suggesting minimal impact of the increased surface area of powder samples.

**Figure 4:**
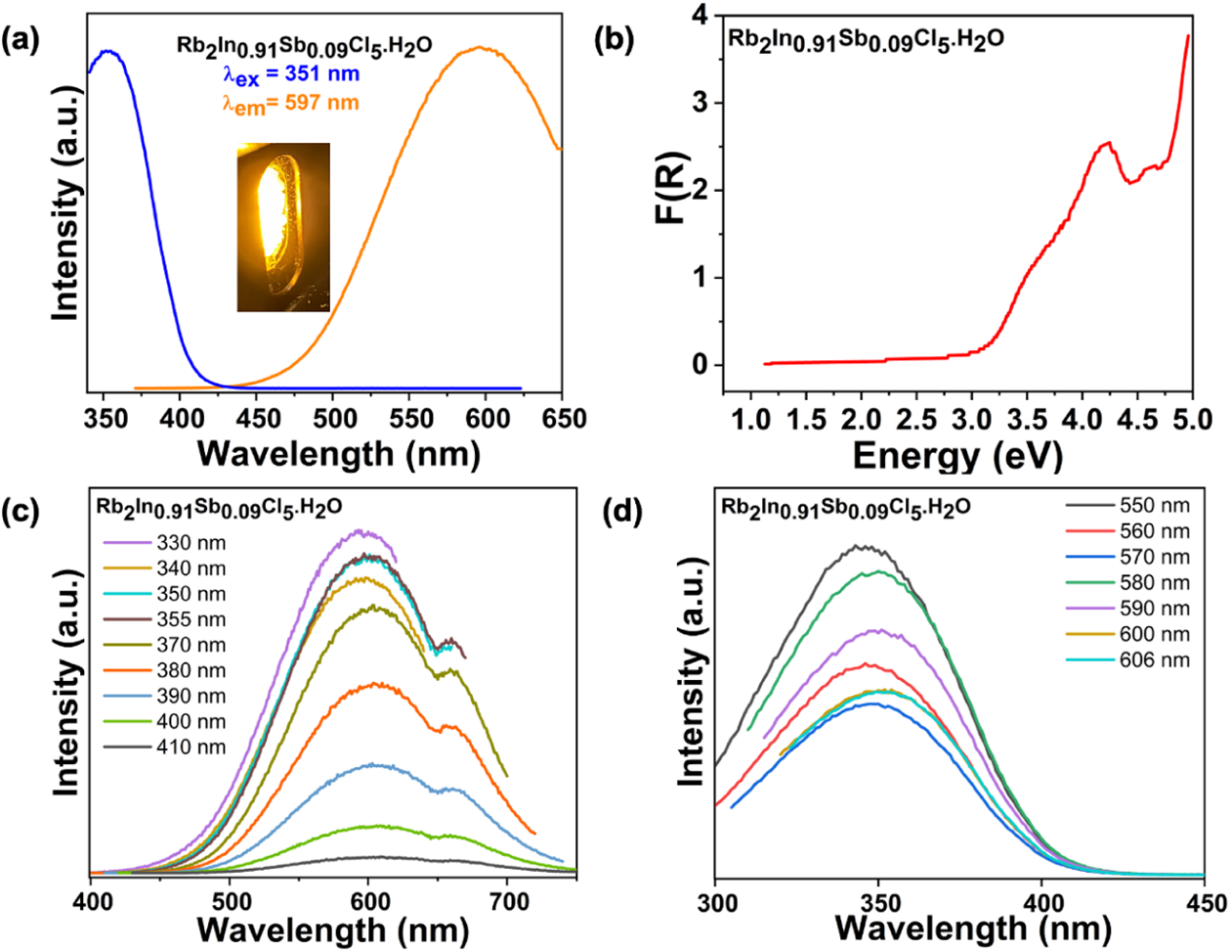
(a) PLE (blue) and PL (orange) spectra for Rb_2_In_0.91(0.2)_Sb_0.09_Cl_5_·H_2_O recorded at room temperature. (b) Diffuse reflectance spectrum converted to Kubelka–Munk function (*F*(*R*)) plotted against photon energy. (c) Excitation-dependent PL spectra and (d) emission-dependent PLE spectra for Rb_2_In_0.91(0.2)_Sb_0.09_Cl_5_·H_2_O.

TGA and DSC measurements were performed on Rb_4_CuSb_2_Cl_11_ and Rb_2_In_0.91(0.2)_Sb_0.09_Cl_5_·H_2_O to evaluate their thermal behavior and decomposition characteristics (Figure 5). For Rb_4_CuSb_2_Cl_11_, the results indicate that the material does not show any decomposition up to 200 °C. The sample evaporation starts above 230 °C, with a sharp first decomposition event observed at 264.1 °C. This weight loss marks the beginning of structural degradation. Following this, additional weight losses were recorded between 344 °C and 360 °C, resulting in a further loss of about 12.1%. These observations were further confirmed by observing a crystal on heating; the crystal shows a change of color from yellow to brown at ∼230 °C. At 250 °C, a clear melting transition was observed, with higher temperature affording a completely dark material. Interestingly, this decomposition pattern closely resembles that reported for Cs_4_CuSb_2_Cl_12_.^20^ These sequential decomposition steps suggest a multistage thermal degradation process. The TGA–DSC data for Rb_2_In_0.91(0.2)_Sb_0.09_Cl_5_·H_2_O reveal an initial weight-loss onset at approximately 155 °C, corresponding to the release of coordinated water molecules from the lattice. A gradual weight loss continues with additional thermal events observed near 297 and 371 °C, which can be attributed to progressive decomposition and eventual structural collapse of the halide framework. This thermal behavior is consistent with previously reported Sb-doped all-inorganic indium halides.^68^

**Figure 5:**
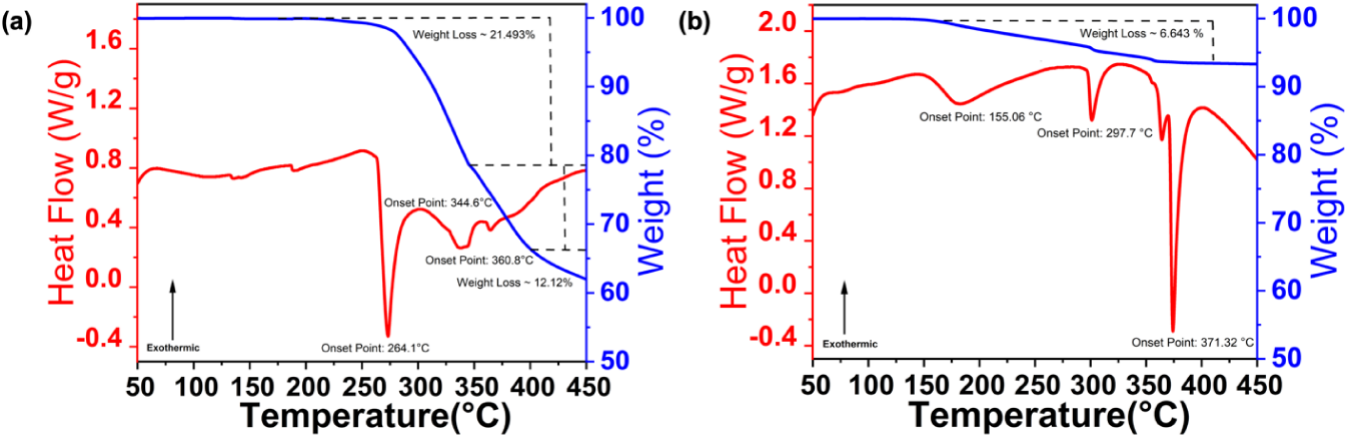
TGA and DSC measurement results for (a) Rb_4_CuSb_2_Cl_11_ and (b) Rb_2_In_0.91(0.2)_Sb_0.09_Cl_5_·H_2_O.

In the literature, all-inorganic Cu­(I) metal halides have received significant research attention due to their promise in electrical, optical, and X-ray radiation detection applications.^69–72^ Although Rb_4_CuSb_2_Cl_11_ exhibits weak light emission properties, making it unsuitable for use as a scintillator material, we assessed its potential as a semiconductor for direct X-ray detection. To check the resistivity, we measured a current–voltage (*I*–*V*) curve using a single crystal of Rb_4_CuSb_2_Cl_11_ (Figure 6a). The material exhibits a resistivity of 1.29 × 10^10^ Ω·cm, which is favorable for minimizing detector leakage current. This value is comparable to those reported for other 0D metal halides, such as MA_3_Bi_2_I_9_ (3.75 × 10^10^ Ω·cm),^73^ (R/S-PPA)_2_BiI_5_ (2.96 × 10^10^ Ω·cm),^74^ and Rb_23_Bi^III^_7_Sb^V^_2_Cl_54_ (1.0 × 10^10^ Ω·cm),^31^ as well as for the two-dimensional metal halides [Cu­(O_2_C–CH_2_–NH_2_)_2_]­Pb_2_Br_4_(1.44 × 10^10^ Ω·cm).^75^ We further determined the *n*_trap_of the Rb_4_CuSb_2_Cl_11_ single crystal using the space-charge-limited-current (SCLC) measurements. Figure 6b shows the SCLC curves, where three distinct current transition regimes are observed. These three regimes correspond to the Ohmic (*I*∝*V*), trap-filled-limited (*I*∝*V* ^*n*^), and Child (*I*∝*V* ^2^). The trap-filled-limited (TFL) regime is characterized by the complete filling of defect traps by charge carriers injected from the metal contacts.^76^ Therefore, the trap states can be determined from the onset voltage *V*_TFL_ of this regime using the following formula,^77^

$$n_{\mathrm{trap}}=\frac{2{\varepsilon \varepsilon }_{0}}{{\mathrm{eL}}^{2}}V_{\mathrm{TFL}}$$

**Figure 6:**
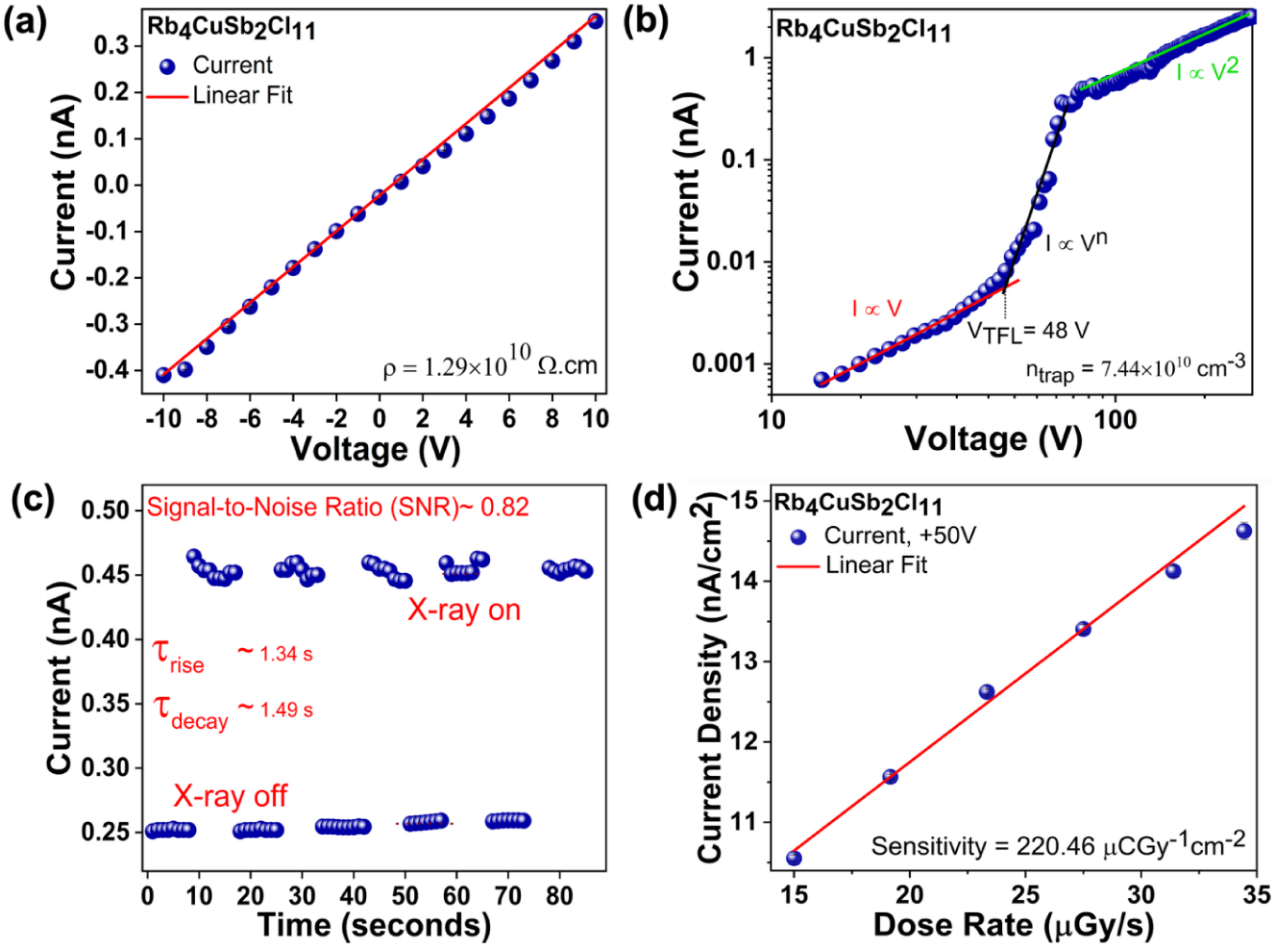
(a) Current–voltage (*I*–*V*) data measured for Rb_4_CuSb_2_Cl_11_. (b) Space-charge-limited-current measurement to estimate the *n*_trap_ levels for Rb_4_CuSb_2_Cl_11_ single crystals. (c) X-ray “on” and “off” current response measurement. (d) X-ray detection sensitivity measurement for Rb_4_CuSb_2_Cl_11_.

Here, *n*_trap_ represents the density of trap states (cm^–3^), ε_0_ is the vacuum permittivity, *ε* is the dielectric constant (= 14.03), *e* denotes the electronic charge, *L* is the crystal thickness, and *V*_TFL_ (= 48 V) is the onset voltage of the TFL regime. The n_trap_for Rb_4_CuSb_2_Cl_11_ is estimated to be 7.44 × 10^10^ cm^–3^, which is comparable to values reported for other metal halides, such as 10^9^–10^10^ cm^–3^ for MAPbBr_3_ and 3.33 × 10^10^ cm^–3^ for Rb_4_Ag_2_BiBr_9_.^78,79^ Following this, a prototype X-ray detector was fabricated using a single crystal of Rb_4_CuSb_2_Cl_11_ to demonstrate its potential for X-ray detection applications. In this experiment, the detector was exposed to soft X-rays generated from a Cu X-ray source. Figure 6c presents the current response recorded during X-ray “on” and “off” measurements. This measurement demonstrates that the Rb_4_CuSb_2_Cl_11_ compound is responsive to soft X-ray photons. The signal-to-noise ratio (SNR), calculated as SNR = $\frac{I_{X-\mathrm{ray on}}-I_{X-\mathrm{ray off}}}{I_{X-\mathrm{ray off}}}$ was determined to be approximately 0.82. The rise and decay times of the X-ray-induced current signal were measured around 1.34 and 1.49 s, respectively, based on the 10% to 90% range of the current amplitude. A slight decrease in current over the measurement period was also observed, which can be attributed to halide ion migration. This behavior is commonly reported in halide-based semiconductors. Furthermore, the fabricated detector was used to determine the detection sensitivity. As shown in (Figure 6d), the sensitivity of the Rb_4_CuSb_2_Cl_11_ was measured to be 220.46 uCGy^–1^cm^–2^ (at electrical field *E* = 50 V/mm). This value is comparable to that of the recently reported one-dimensional organic–inorganic metal halide [(CH_3_)_3_SO]­Cu_2_I_3_ (200.54 uCGy^–1^cm^–2^ at *E* = 41.67 V/mm), two-dimensional halide Rb_4_Ag_2_BiBr_9_ (222.03 uCGy^–1^cm^–2^ at *E* = 24 V/mm), and BDAPbI_4_ (BDA = NH_3_C_4_H_8_NH_3_) (242 uCGy^–1^cm^–2^ at *E* = 0.31 V/mm).^79–81^

The calculated electronic band structure and PDOS for Rb_4_CuSb_2_Cl_11_ are provided in Figure 7. The band structure (Figure 7a) shows an indirect band gap of 2.98 eV, which agrees with the experimentally estimated value (2.89 eV). The PDOS plots (Figures 7b–e) show the orbital contributions of each atom and their orbitals to the electronic states near the band edges. Notably, the conduction band minimum (CBM) is primarily composed of *p* orbitals from Sb and Cl. A minor contribution from the *s* orbitals of Sb and Cl is also observed near the CBM. In contrast, orbitals of Cu and Rb atoms do not significantly contribute to the CBM. On the other hand, the valence band maximum (VBM) is predominantly composed of the *d* orbitals of Cu and the *p* orbitals of Cl. Among the orbitals from Cu, *d*_*xy*_ and $d_{x^{2}-y^{2}}$ show the strongest contributions near the VBM, with a smaller contribution from *d*_*z*_^2^. Also significant are Cl *p*_*x*_ and *p*_*z*_ orbital contributions to the VBM, indicating the hybridization between *d* orbitals of Cu and *p* orbitals of Cl. Rb has negligible involvement in both the valence and conduction bands, as expected for the *A* cation, which typically act as charge-balancing cations rather than active electronic contributors. Interestingly, the CBM dominated by the *p* orbitals of Sb and Cl suggests that the CBM is localized on [SbCl_4_]^−^ anions. Conversely, the flat VBM dominated by the *d* orbitals of Cu and *p* orbitals of Cl indicates that [CuCl_3_]^2–^ anions localize VBM. As a result, an indirect band gap is observed for Rb_4_CuSb_2_Cl_11_. The observed optical transitions result in a charge transfer between structurally separate [CuCl_3_]^2–^ and [SbCl_4_]^−^ anions, leading to the observed weak PL properties.

**Figure 7:**
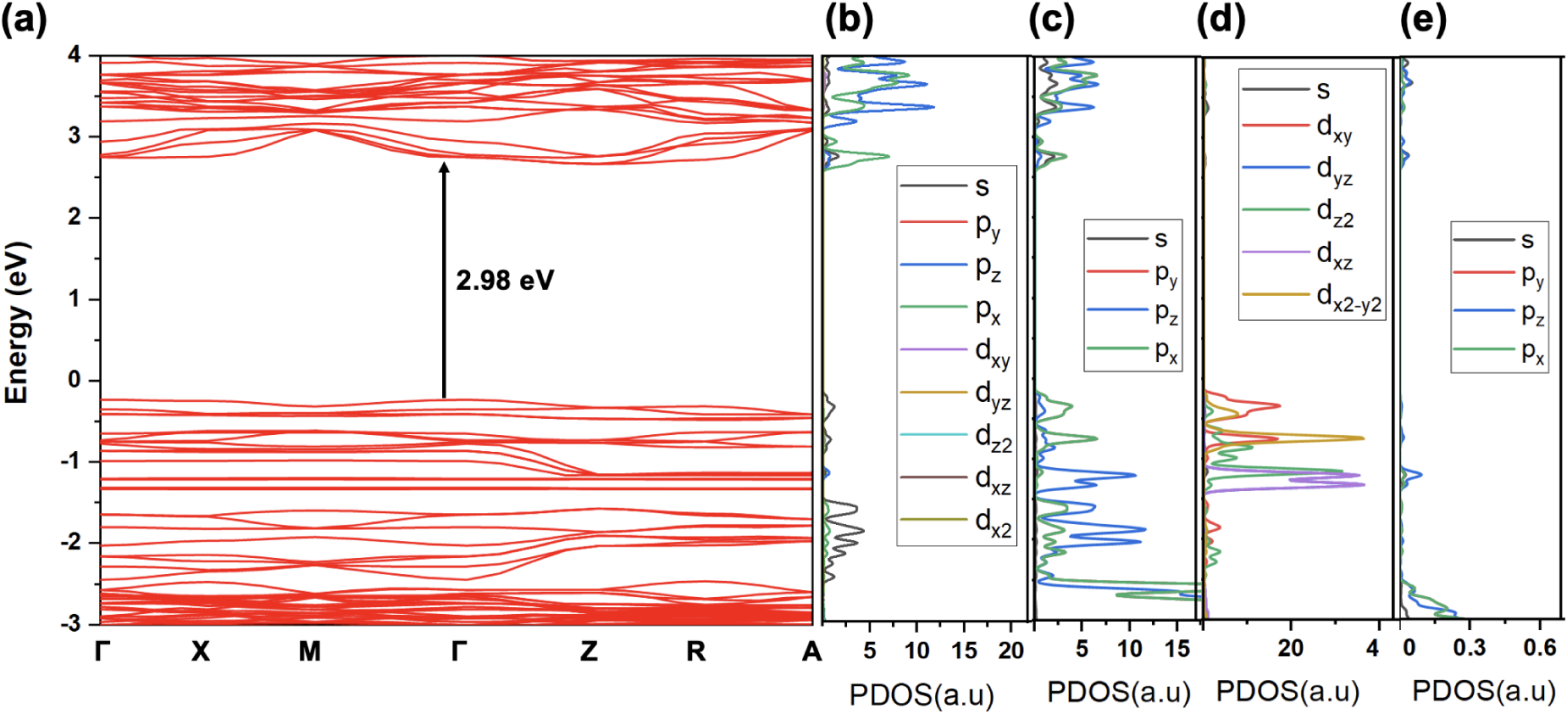
(a) Computed band structure of Rb_4_CuSb_2_Cl_11_. PDOS (each orbital) of (b) Sb, (c) Cl, (d) Cu, and (e) Rb. Note that PDOS scale for Rb is very small as compared to others.

To investigate the effect of Sb doping in Rb_2_InCl_5_·H_2_O, we performed similar DFT studies on the parent Rb_2_InCl_5_·H_2_O and hypothetical Rb_2_SbCl_5_·H_2_O (obtained by replacing In with Sb). The electronic band gaps of Rb_2_InCl_5_·H_2_O and Rb_2_SbCl_5_·H_2_O, along with the PDOS for each element, are shown in Figure 8. The calculated band gaps are 4.85 eV for Rb_2_InCl_5_·H_2_O and 2.82 eV for Rb_2_SbCl_5_·H_2_O, indicating that Sb incorporation significantly reduces the band gap. The PDOS analysis reveals that Sb contributes prominently through its *p* orbitals near the CBM and its *s* orbital near the VBM. These Sb states also hybridize with the *p* orbitals of Cl near the CBM. Consequently, Sb doping shifts the valence band upward, effectively reducing the band gap and modifying the electronic structure. In particular, the interaction between the Sb *s* band and the Cl *p* band appears to generate states near the band edges (see Figure 8h as compared with Figure 8c), further lowering the band gap. Overall, this analysis suggests that Sb doping reduces the band gap by introducing states near both the conduction and valence band edges.

**Figure 8:**
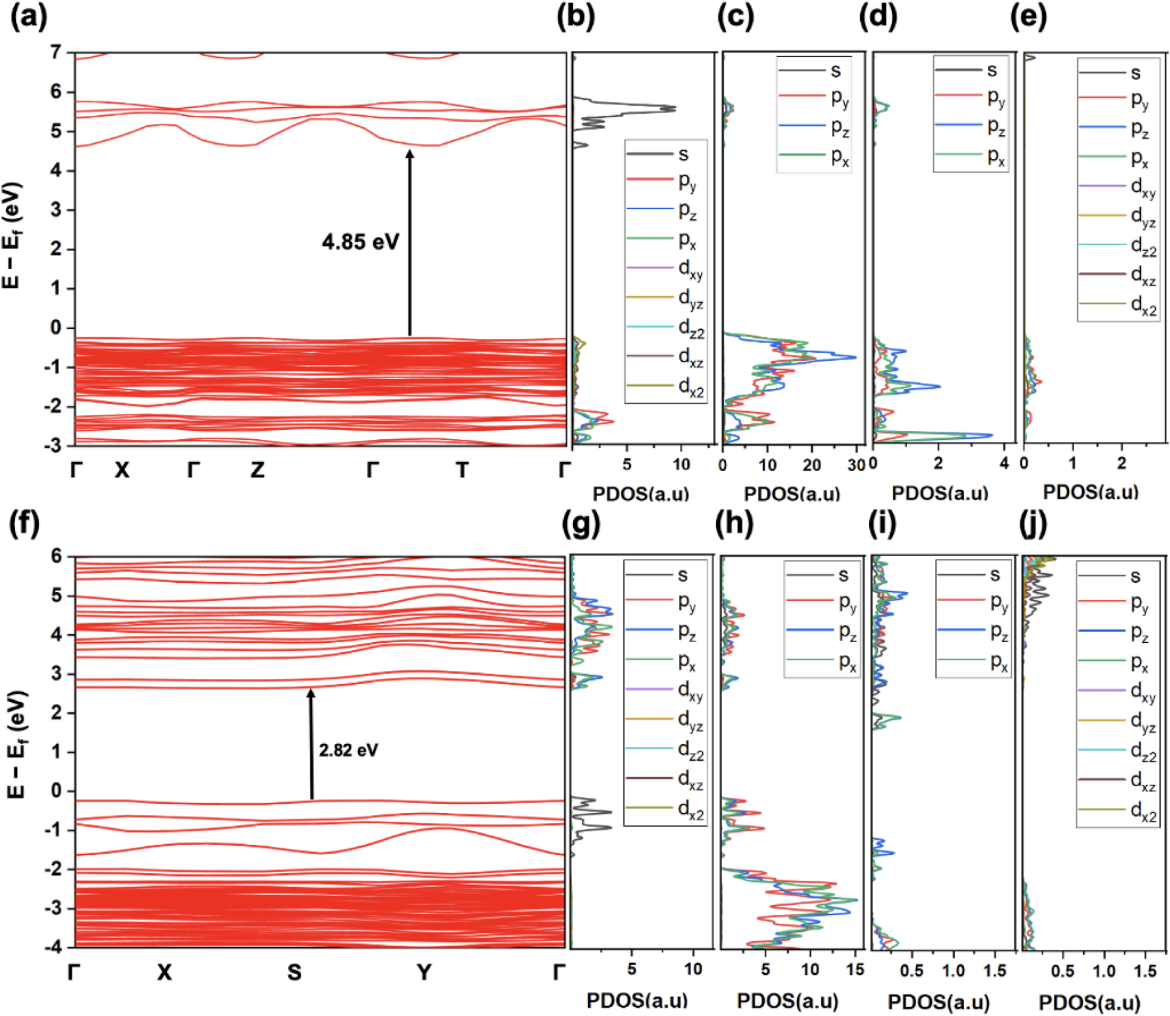
Electronic band structures and Projected Density of States (PDOS) plots for Rb_2_InCl_5_·H_2_O (top, a–e) and Rb_2_SbCl_5_·H_2_O (bottom, f–j). PDOS plots showing contributions of (b) In, (g) Sb (c, h) Cl and (d, i) O and (e, j) Rb. Note that PDOS scale is not the same for all elements.

## 4 Conclusions

In summary, we have synthesized a new lead-free wide band gap semiconductor Rb_4_CuSb_2_Cl_11_, which is the first report reported quaternary compound in the Rb–Cu–Sb–Cl system. Rb_4_CuSb_2_Cl_11_ crystallizes in a tetragonal system with a centrosymmetric space group *P4*_*2*_ */m*; this compound has a new structure type featuring isolated [SbCl_4_]^−^ seesaw and [CuCl_3_]^2–^ trigonal planar units. These molecular anions are separated by Rb^+^ cations giving rise to a unique 0D structure. Optical characterization and DFT calculations reveal an indirect band gap of 2.89 eV. PL measurements show that the emission is quenched. The quenched PL is attributed to the unique electronic structure of the material including the indirect nature of the band gap, and VBM and CBM that are primarily derived from Cu and Sb orbitals, respectively. This Cu­(I) → Sb­(III) charge transfer and the separation of charges on distant structural units contributes to the quenching of luminescence in this material. Unlike related Cu­(I) and Sb­(III) with prospective scintillators applications, Rb_4_CuSb_2_Cl_11_ could be employed as a semiconductor in qualitative X-ray detection applications. Preliminary electrical measurements indicate that the material has resistivity and a *n*_trap_comparable to that of hybrid lead halides.

Additionally, we investigated the optical properties of Rb_2_In_0.91(0.2)_Sb_0.09_Cl_5_·H_2_O, which was obtained when targeting substitutional derivatives of Rb_4_CuSb_2_Cl_11_. The initial SCXRD refinement suggested lattice parameters consistent with that of Rb_2_SbCl_5_O, a compound previously reported as an impurity phase from a reaction mixture also containing In­(III).^31^ However, our subsequent SEM–EDS, FTIR, and careful structural analysis confirmed the correct composition as Rb_2_In_0.91(0.2)_Sb_0.09_Cl_5_·H_2_O. XPS verified the presence of both In^3+^ and Sb^3+^, and the PL of the compound is consistent with the 5s^2^ lone-pair driven emission of Sb­(III) centers.^82^ The compound exhibits broadband yellowish-orange emission with a moderately high PLQY of 18%. Our experimental and computational results demonstrate that mixed Sb­(III) substitution into In­(III) halides effectively tunes the photophysical behavior including band gaps and photoemission properties.

## Supplementary Material



## Notes

Crystal structure, including fractional atomic coordinates, bond lengths and angles, simulated and experimental PXRD patterns, other structural features, Tauc plots, XPS spectra, temperature-dependent PL, SEM–EDS, and FTIR spectrum (PDF)
