# Supplementary material for: Rb4CuSb2Cl11 and Rb2In0.91(0.2)Sb0.09Cl5·H2O: Wide Band Gap 0D Metal Halide Semiconductors
Source: Inorg Chem. 2025 Dec 16;65(1):403–15. doi: 10.1021/acs.inorgchem.5c04529 (PMC12801379; doi:10.1021/acs.inorgchem.5c04529)
Supplement: Supplementary file 1 [file ic5c04529_si_001.pdf]

Supplementary Information

$Rb_4CuSb_2Cl_{11}$ and $Rb_2In_{0.91(0.2)}Sb_{0.09}Cl_5·H_2O$: Wide band gap 0D metal halide semiconductors

Hamza Shoukat,[1] Tamanna Pinky,[1] Muhammad S. Muhammad,[1] Zaheer Masood,[2] Arslan Akbar,[3] Nobuyuki Yamamoto,[1] Sharad Puri,[4] David N. McIlroy,[4] Bin Wang,[2] Jakoah Brgoch,[3] Bayram Saparov[1*]

[1]*Department of Chemistry & Biochemistry*, *University of Oklahoma, Norman, OK, USA*

[2]*School of Sustainable Chemical, Biological and Materials Engineering, University of Oklahoma, Norman, OK 73019, USA*

[3]*Department of Chemistry and Texas Center for Superconductivity, University of Houston, Houston, Texas 77204, USA*

[4]*Department of Physics, Oklahoma State University, Stillwater, Oklahoma 74078, USA*

*Author to whom correspondence should be addressed: saparov@ou.edu

Table S1. Fractional atomic coordinates and equivalent isotropic displacement parameters (U_{eq}^{a}) for $\mathbf{Rb_4CuSb_2Cl_{11}}$.

Atom	*x*	*y*	*z*	U_{eq}, (Å^2)
		$\mathbf{Rb_4CuSb_2Cl_{11}}$		
Rb1	0.000000	1.000000	0.250000	0.0465(4)
Rb2	0.000000	0.500000	0.25587(11)	0.0412(3)
Rb3	0.23242(7)	0.26721(6)	0.77963(9)	0.0432(2)
Rb4	0.500000	0.500000	0.750000	0.0400(4)
Cu1	0.51136(11)	0.75889(11)	0.500000	0.0515(5)
Sb1	0.25345(5)	1.03897(5)	0.500000	0.02201(19)
Sb2	0.25538(5)	0.48918(5)	0.500000	0.02124(19)
Cl1	0.24943(15)	1.02657(16)	0.26701(18)	0.0374(5)
Cl2	0.1057(2)	1.1313(2)	0.500000	0.0421(8)
Cl3	0.25070(15)	0.50351(16)	0.73334(18)	0.0378(5)
Cl4	0.10556(19)	0.3932(2)	0.500000	0.0381(7)
Cl5	0.4139(2)	0.8902(2)	0.500000	0.0360(6)
Cl6	0.1573(2)	0.6333(2)	0.500000	0.0366(6)
Cl7	0.4166(2)	0.6174(2)	0.500000	0.0432(7)
Cl8	0.67096(18)	0.7410(2)	0.500000	0.0371(6)
Cl9	0.1538(2)	0.89416(19)	0.500000	0.0332(6)

$^{a}U_{eq}$ is defined as one-third of the trace of the orthogonalized U_{ij} tensor.

Table S2. Fractional atomic coordinates and equivalent isotropic displacement parameters (U_{eq}[a]) for **$Rb_2In_{0.91(0.2)}Sb_{0.09}Cl_5{\cdot}H_2O$**.

Atom	*x*	*y*	*z*	U_{eq}, (Å^2)
		**$Rb_2In_{0.91(0.2)}Sb_{0.09}Cl_5{\cdot}H_2O$**		
In1	0.38589(6)	0.2500	0.31011(12)	0.0138(5)
Sb1	0.38589(6)	0.2500	0.31011(12)	0.0138(5)
Rb1	0.35573(8)	0.00067(10)	0.84468(15)	0.0305(6)
Cl1	0.2477(2)	0.2500	0.0974(4)	0.0228(8)
Cl2	0.3960(2)	0.4961(2)	0.3209(4)	0.0280(8)
Cl3	0.4967(2)	0.2500	0.0427(5)	0.0289(9)
Cl4	0.2772(3)	0.2500	0.5789(5)	0.0286(9)
O1	0.5092(8)	0.2500	0.5064(16)	0.035(3)
H1A	0.4663	0.2238	0.5781	0.053
H1B	0.5282	0.3201	0.5377	0.053

[a]U_{eq} is defined as one-third of the trace of the orthogonalized U_{ij} tensor.

Table S3. Selected bond distances and angles for $\mathbf{Rb_4CuSb_2Cl_{11}}$ and $\mathbf{Rb_2In_{0.91(0.2)}Sb_{0.09}Cl_5{\cdot}H_2O}$.

Atom pair	Distance (Å)	Label	Angle (°)
		$\mathbf{Rb_4CuSb_2Cl_{11}}$	
Sb1 – Cl1	2.619(2)	Cl1 – Sb1 – Cl1	172.15(10)
Sb1 – Cl2	2.395(3)	Cl2 – Sb1 – Cl1	90.95(5)
Sb1 – Cl9	2.416(3)	Cl2 – Sb1 – Cl9	87.45(9)
Sb2 – Cl3	2.625(2)	Cl9 – Sb1 – Cl1	86.24(5)
Sb2 – Cl4	2.446(3)	Cl3 – Sb2 – Cl3	170.95(10)
Sb2 – Cl6	2.396(3)	Cl4 – Sb2 – Cl3	91.14(5)
Cu1 – Cl5	2.247(3)	Cl3 – Sb2 – Cl7	88.42(5)
Cu1 – Cl7	2.340(3)	Cl8 – Cu1 – Cl5	132.99(13)
Cu1 – Cl8	2.208(3)	Cl8 – Cu1 – Cl7	117.39(13)
		$\mathbf{Rb_2In_{0.91(0.2)}Sb_{0.09}Cl_5{\cdot}H_2O}$	
In1 – Cl1	2.482(4)	Cl4 – In1 – Cl2	90.58(7)
In1 – Cl2	2.514(3)	O1 – In1 – Cl2	86.37(7)
In1 – Cl3	2.489(4)	Cl4 – In1 – Cl3	179.28(14)
In1 – Cl4	2.479(4)	Cl4 – In1 – Cl1	90.46(12)
In1 – O1	2.244(11)	Cl3 – Sb1 – Cl2	89.38(7)
O – H	0.8402	O1-In1-Cl1	179.0(3)

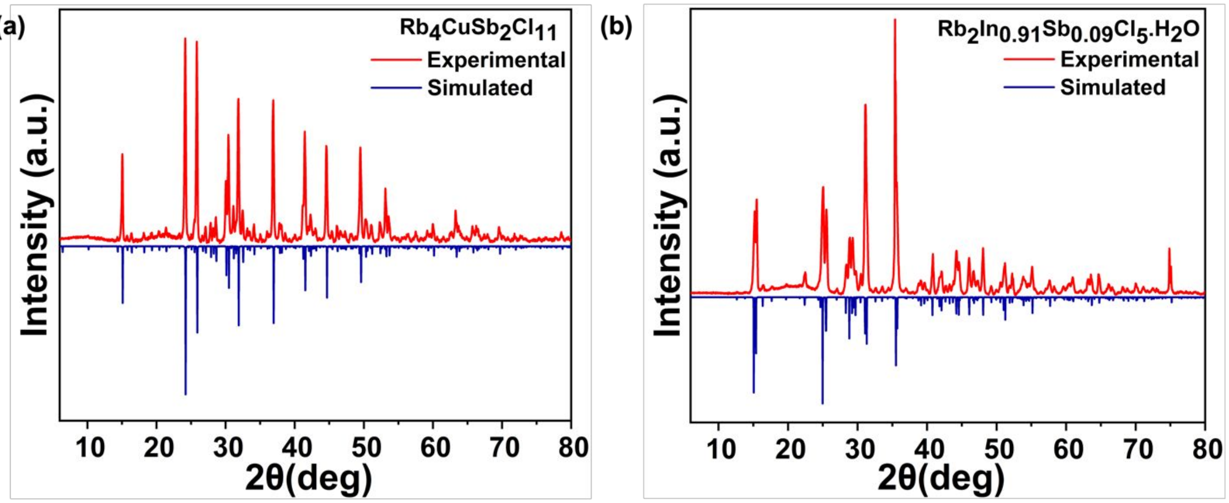

Figure S1. Simulated and experimental XRD patterns for (a) $Rb_4CuSb_2Cl_{11}$ and (b) $Rb_2In_{0.91(0.2)}Sb_{0.09}Cl_5{\cdot}H_2O$.

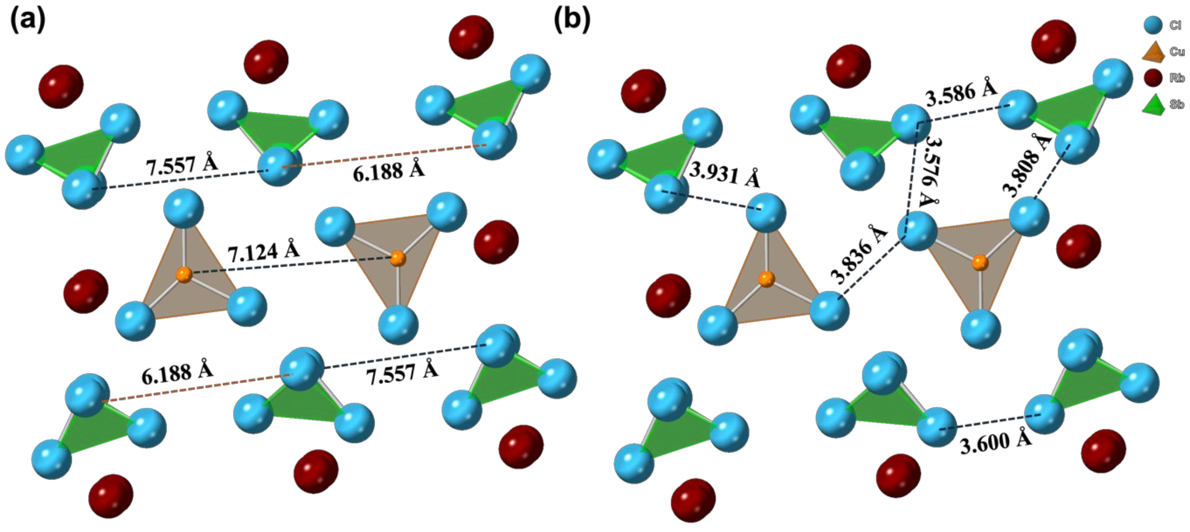

Figure S2. (a) Interatomic Cu···Cu and Sb···Sb distances along different crystallographic directions. (b) Adjacent trigonal planar $[CuCl_3]^{2-}$ and see-saw $[SbCl_4]^{-}$ units exhibit distinct Cl···Cl contacts within the asymmetric unit of $Rb_4CuSb_2Cl_{11}$.

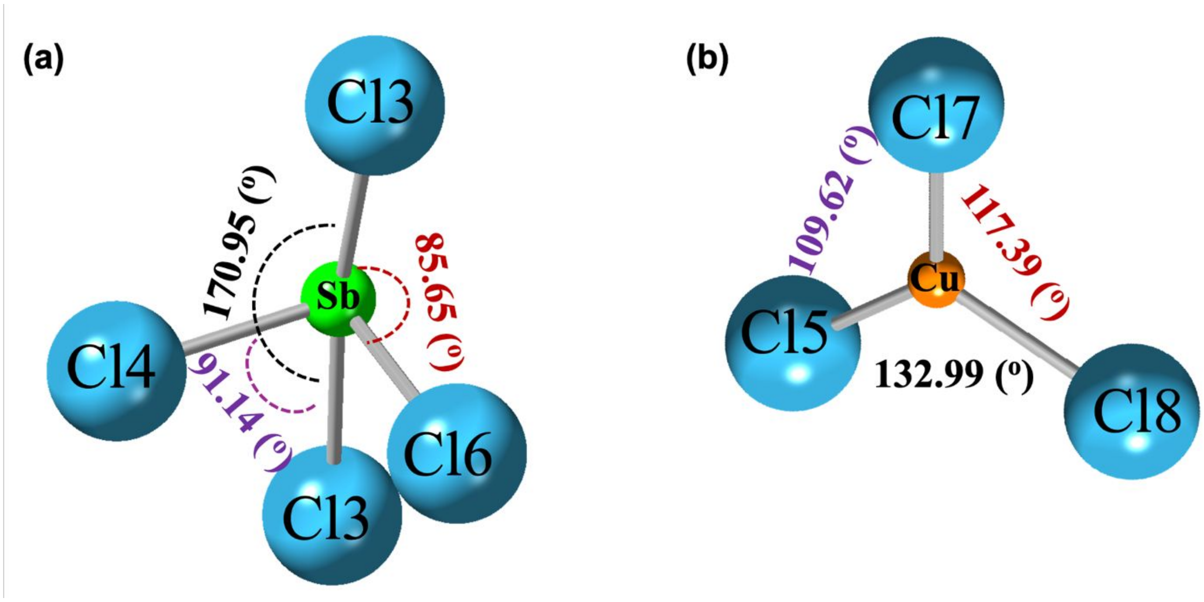

Figure S3. (a) Cl---Sb---Cl bond angles within the see-saw $[SbCl_4]^-$ anions. (b) Cl---Cu---Cl bond angles within the distorted trigonal planar $[CuCl_3]^{2-}$ anions.

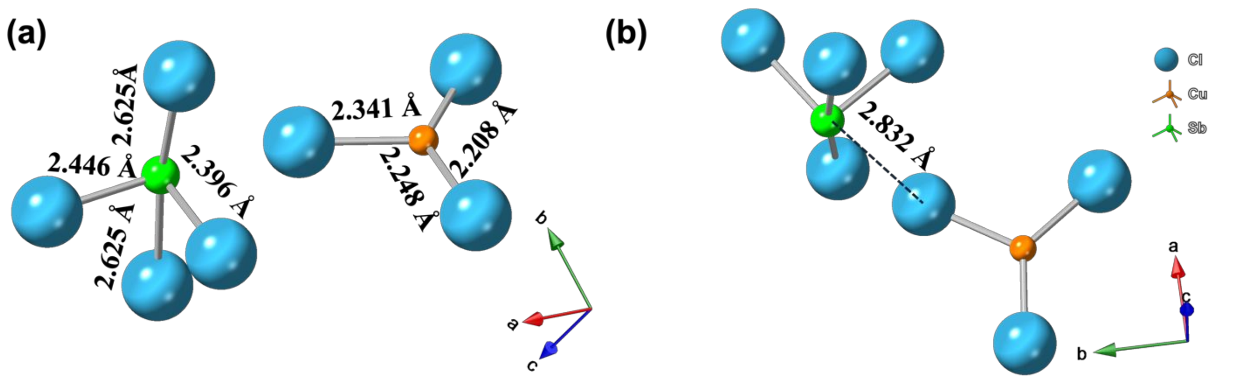

Figure S4. (a) Cu—Cl and Sb—Cl bond lengths within the trigonal planar $[CuCl_3]^{2-}$ and see-saw $[SbCl_4]^-$ anions. (b) The see-saw $[SbCl_4]^-$ anions are capped by chlorides on the adjacent $[CuCl_3]^{2-}$ anions via an elongated Sb···Cl contact yielding a distorted square pyramidal geometry.

Rb Coordination #	Rb coordination environment in $Rb_4CuSb_2Cl_{11}$
Rb1 = 12	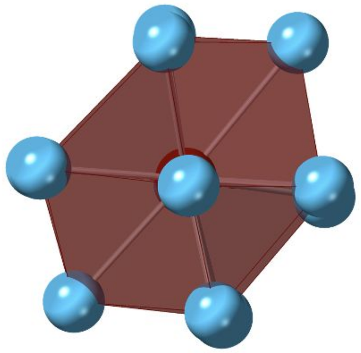
Rb2 & Rb4 = 8	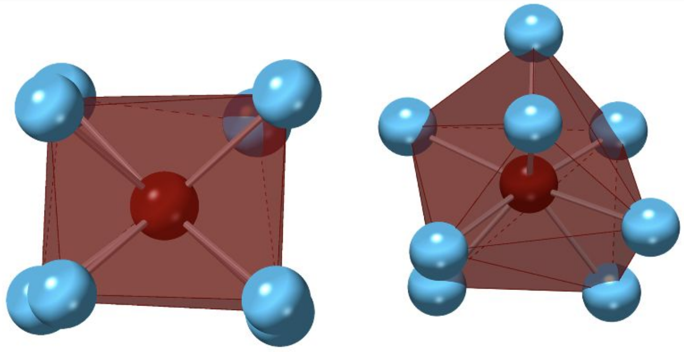
Rb3 = 9	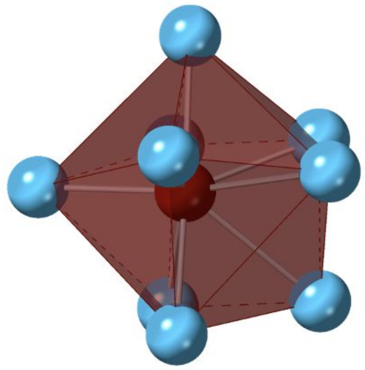

Figure S5. There are four crystallographically unique Rb sites with different coordination environments within the asymmetric unit of $Rb_4CuSb_2Cl_{11}$.

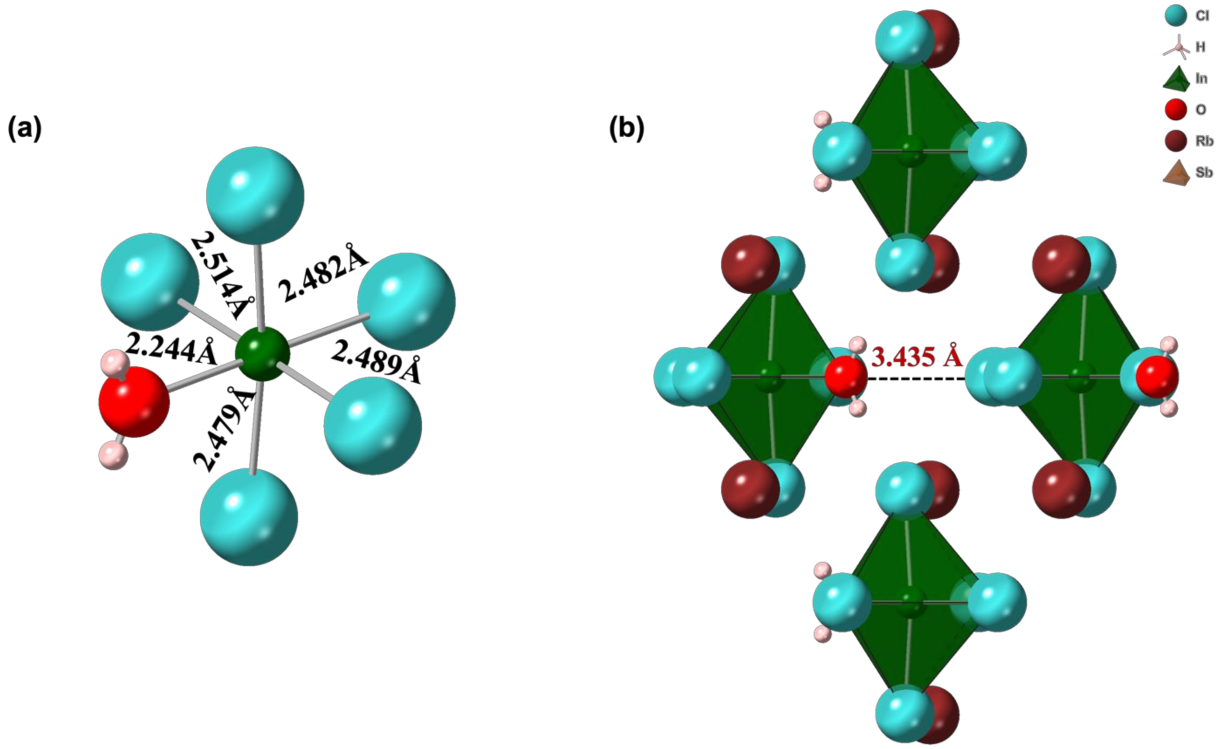

Figure S6. (a) The $[InCl_5H_2O]^{2-}$ octahedra are distorted due to the dissimilar ligands around the In-center in $Rb_2In_{0.91(0.2)}Sb_{0.09}Cl_5{\cdot}H_2O$. (b) The O···Cl distances between adjacent octahedra indicate both separation and close packing in the structure of the vacancy-ordered perovskite $Rb_2In_{0.91(0.2)}Sb_{0.09}Cl_5{\cdot}H_2O$.

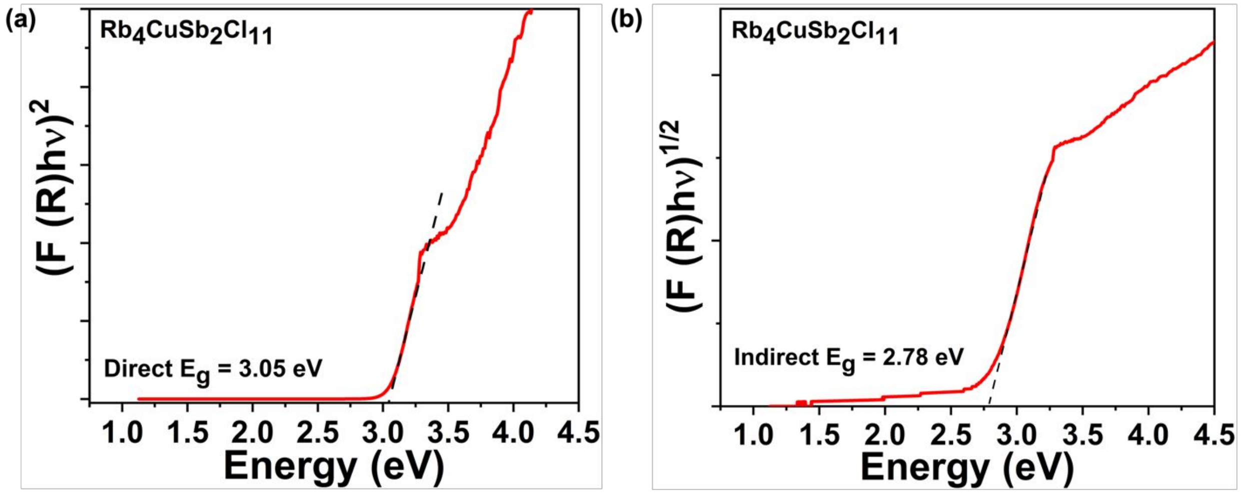

Figure S7. Tauc plots assuming (a) direct and (b) indirect band gaps for $Rb_4CuSb_2Cl_{11}$.

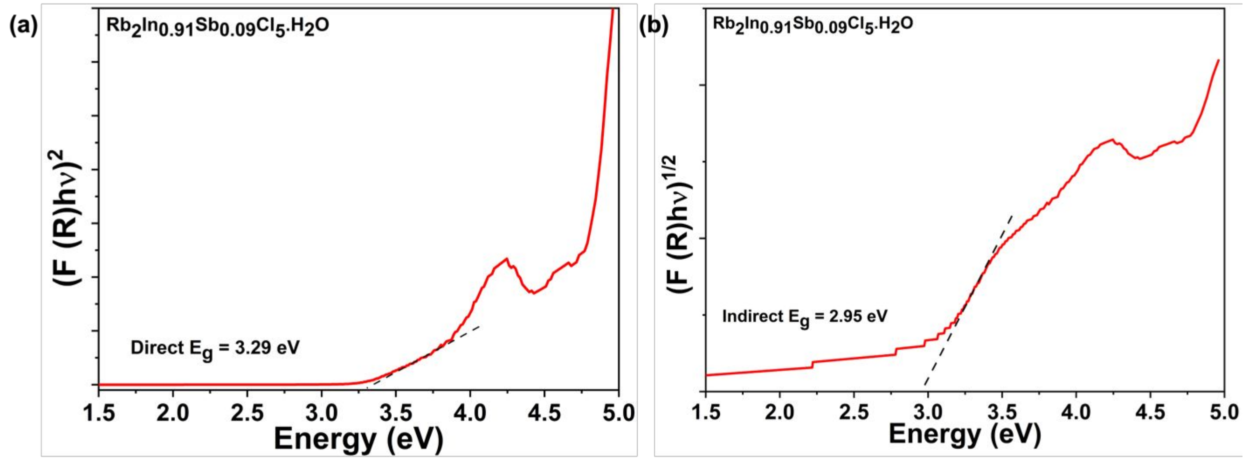

Figure S8. Tauc plots assuming (a) direct and (b) indirect band gaps for $Rb_2In_{0.91(0.2)}Sb_{0.09}Cl_5{\cdot}H_2O$.

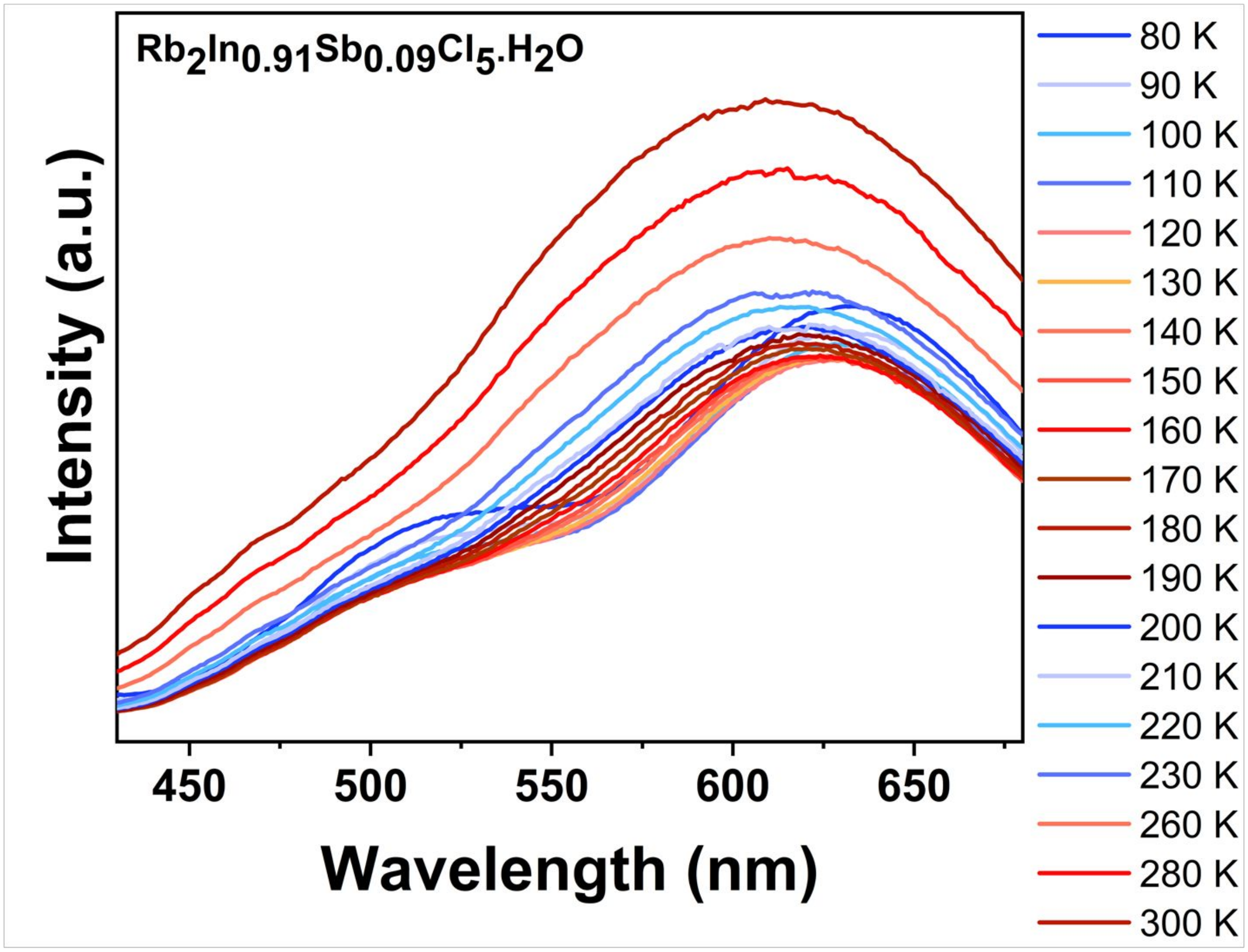

Figure S9. Temperature-dependent photoluminescence spectra for $Rb_2In_{0.91(0.2)}Sb_{0.09}Cl_5{\cdot}H_2O$.

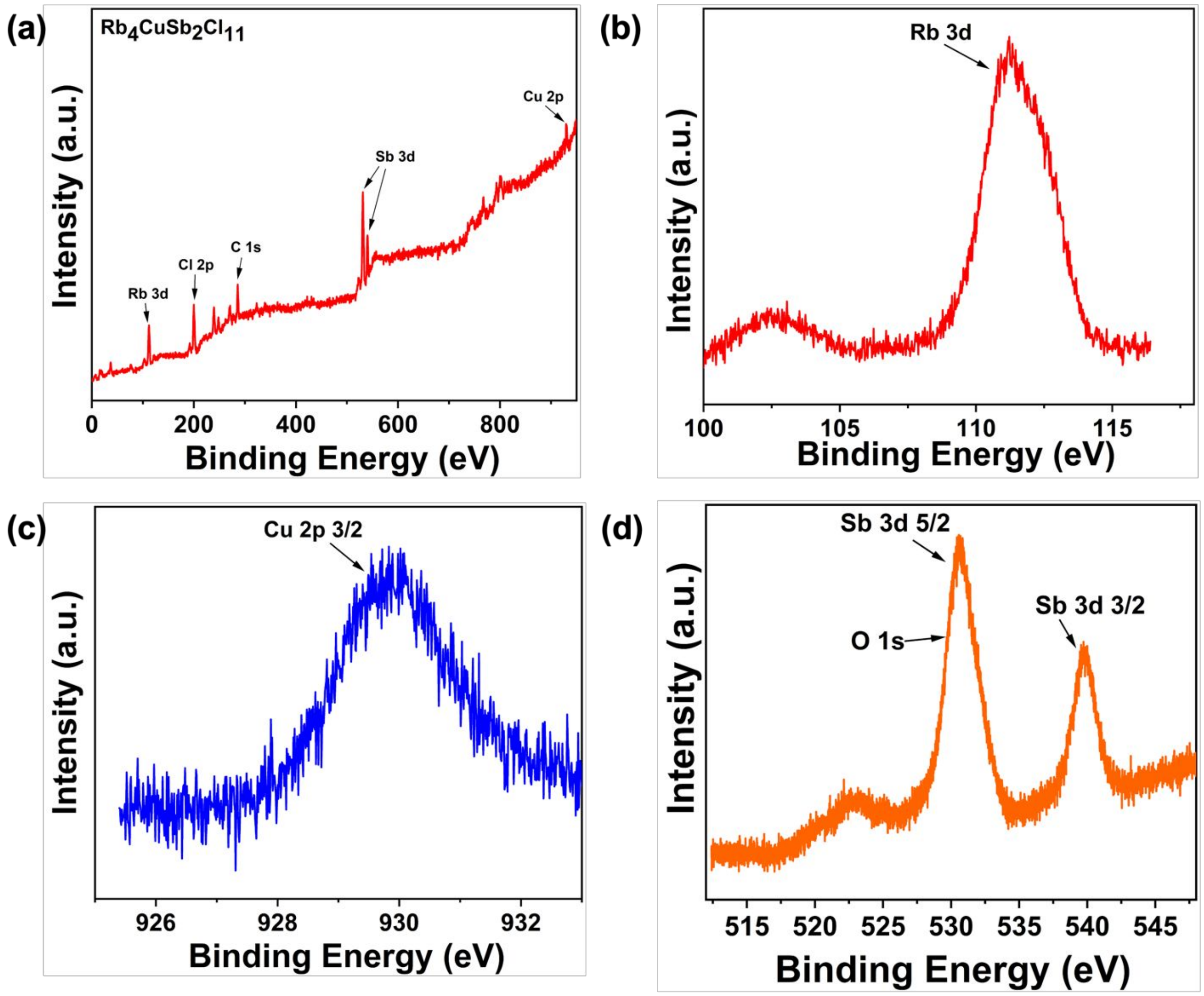

Figure S10. XPS spectra for $Rb_4CuSb_2Cl_{11}$: (a) a survey spectrum, (b) the Rb 3d region, (c) the Cu 2p region, and (d) the Sb 3d region.

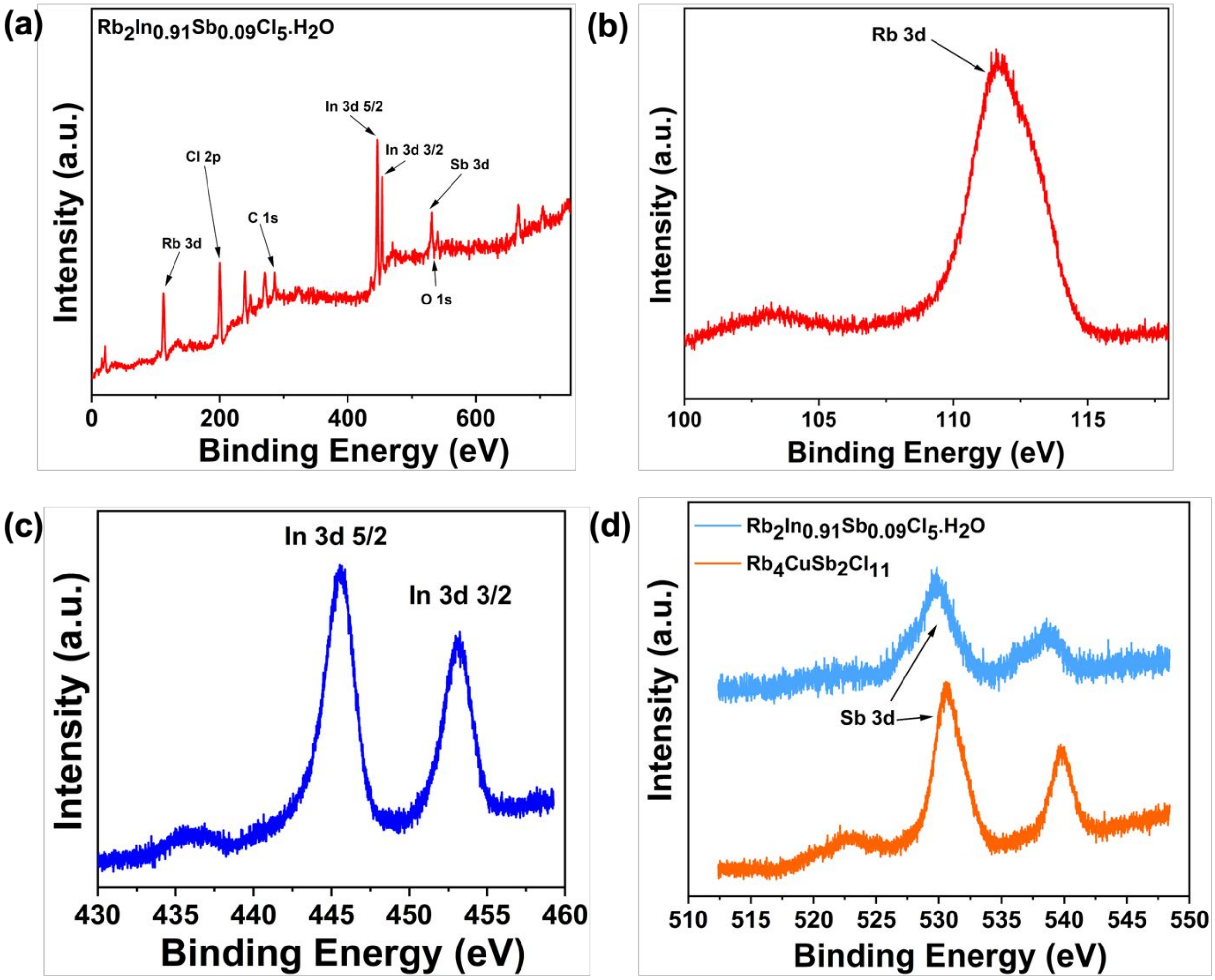

Figure S11. XPS spectra for $Rb_2In_{0.91(0.2)}Sb_{0.09}Cl_5 \cdot H_2O$: (a) a survey spectrum, (b) the Rb 3d region, (c) the In 3d region, and (d) the Sb 3d region of $Rb_2In_{0.91(0.2)}Sb_{0.09}Cl_5 \cdot H_2O$ compared to that of $Rb_4CuSb_2Cl_{11}$.

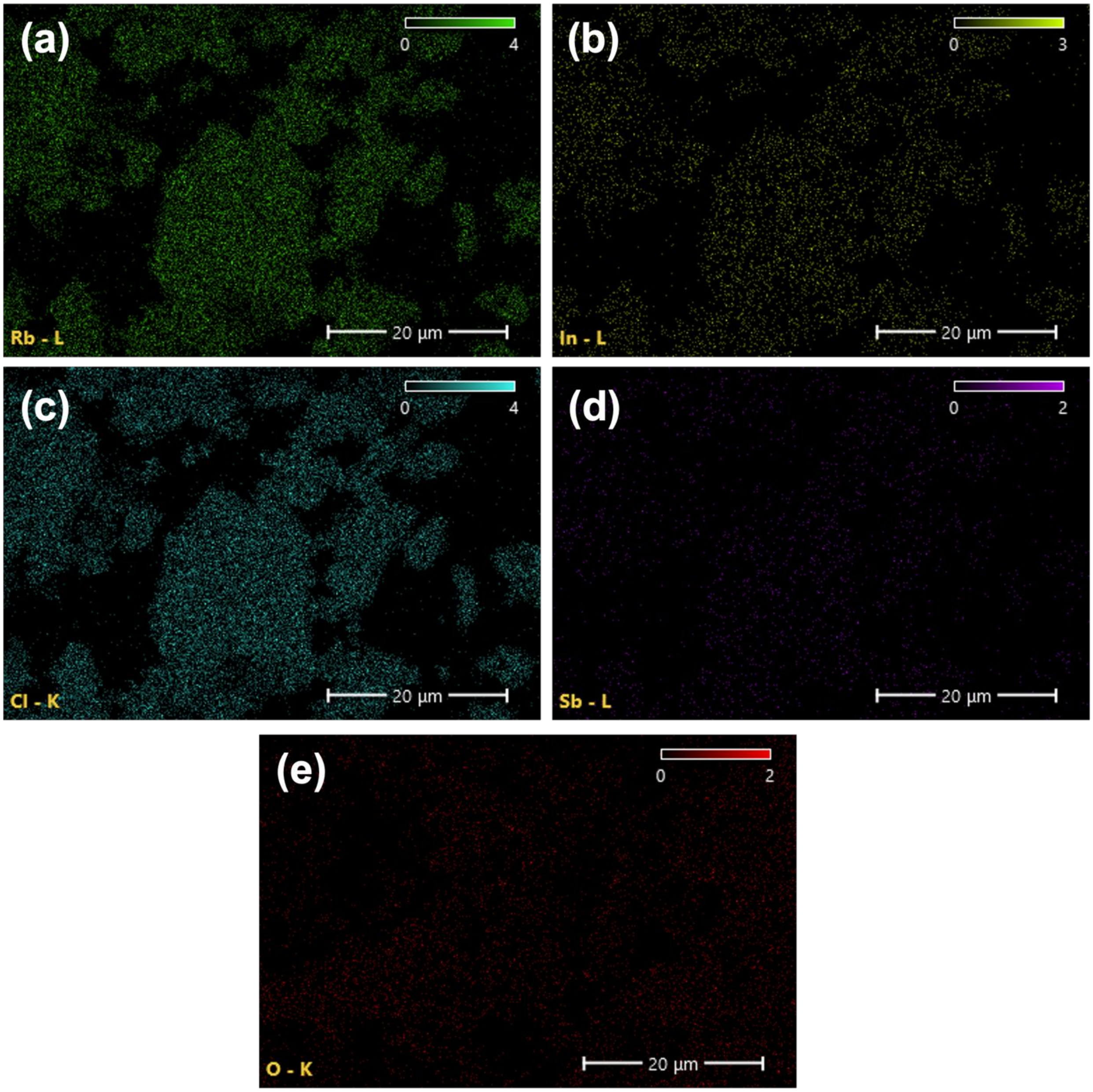

Figure S12. SEM-EDS elemental mapping of $Rb_2In_{0.91(0.2)}Sb_{0.09}Cl_5{\cdot}H_2O$: (a) Rb, (b) In, (c) Cl, (d) Sb, and (e) O.

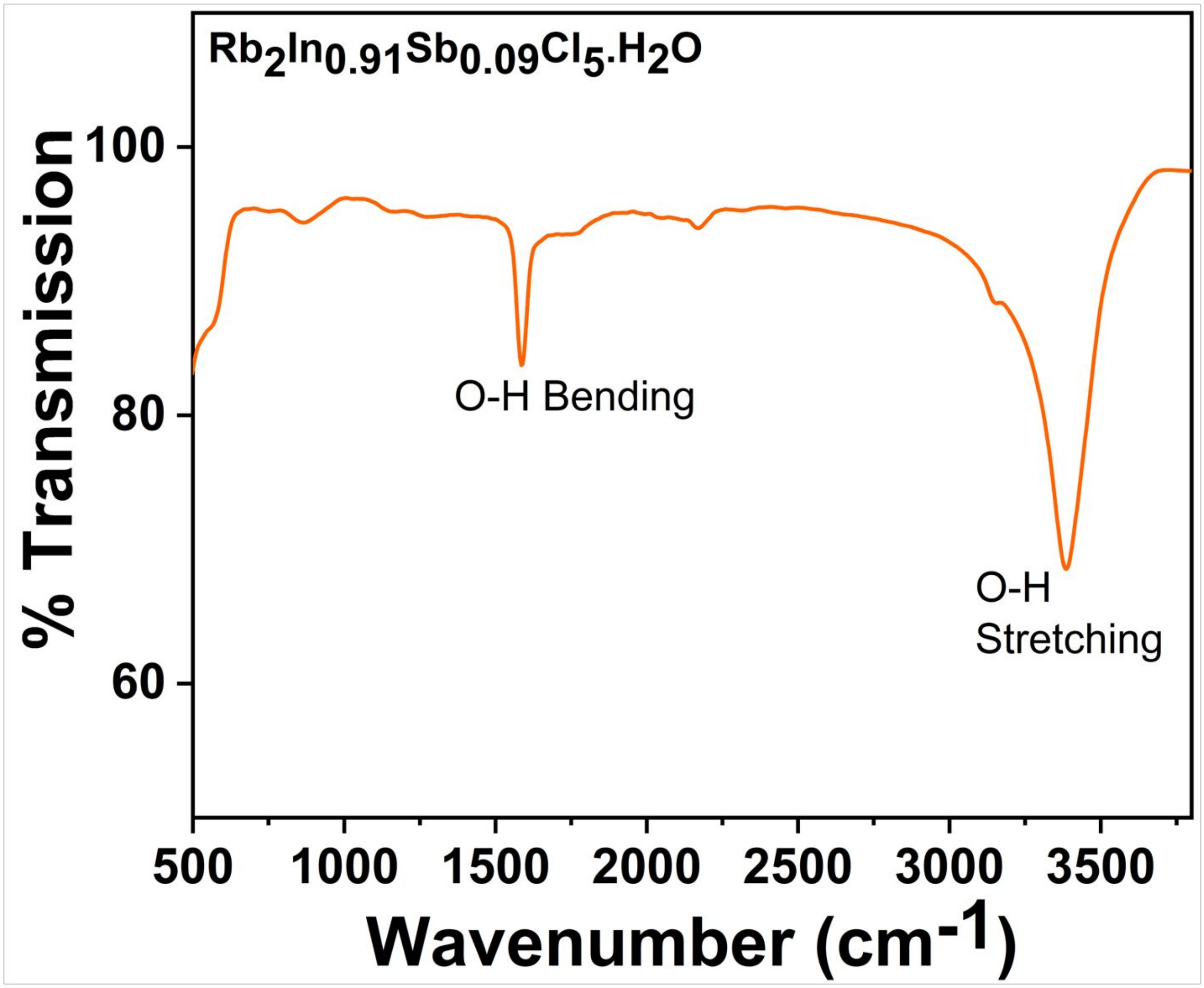

Figure S13. FTIR spectrum showing O-H stretching and bending peaks for $Rb_2In_{0.91(0.2)}Sb_{0.09}Cl_5 \cdot H_2O$.
